# NamiRNA‐Enhancer Network: The Changing Era Challenge and Chance of miRNA

**DOI:** 10.1002/advs.202506830

**Published:** 2025-08-26

**Authors:** Lu Chen, Ying Liang, Hongyan Wei, Mengxing Liu, Jin Wu, Wei Li, Wenxuan Li, Yuxiao Jin, Yinshan Li, Wei Zhao, Min Xiao, Kaicheng Zhou, Shuai Yang, Wenqiang Yu

**Affiliations:** ^1^ Shanghai Public Health Clinical Center and Department of General Surgery Huashan Hospital Cancer Metastasis Institute and Laboratory of RNA Epigenetics Institutes of Biomedical Sciences Shanghai Medical College Fudan University Shanghai 200032 China; ^2^ Department of Pharmacy and Drug Development Center Precision Pharmacy Tangdu Hospital Fourth Military Medical University Xi'an 710038 China; ^3^ Research and Development Department Shanghai Epicurer Biotechnology Co., Ltd Shanghai 200233 China; ^4^ Laboratory Animal Center Fudan University MOE Frontiers Center for Brain Science Fudan University Shanghai 200032 China; ^5^ Ningxia Institution of Clinical Medicine The Third Clinical Medicine College People's Hospital of Ningxia Hui Autonomous Region Ningxia Medical University Yinchuan 750002 China

**Keywords:** enhancer, human identical sequences, NamiRNAs, small nucleotide drugs, transcriptional activation

## Abstract

MicroRNAs (miRNAs) systematically orchestrate multiple biological processes via gene regulation in various organisms. Conventionally, miRNAs bind to untranslated regions of mRNAs to modulate gene silencing in the cytoplasm. Recent studies have revealed that a subset of miRNAs located in the nucleus can efficiently activate gene transcription by targeting enhancers, named as Nuclear Activating miRNAs (NamiRNAs). The fundamental principle of this regulatory pattern is that NamiRNAs as “wedges” open double strands of genomic DNA and provide the prerequisite for RNA polymerase II‐mediated transcription. Specifically, NamiRNAs bind to their target enhancers and form the hybrids of miRNAs and single‐stranded DNA, which are recognized by Argonaute 2 (AGO2) to maintain the single‐stranded state. Meanwhile, AGO2 protects these miRNA‐DNA hybrids from RNase H‐mediated degradation to maintain their stability. Additionally, NamiRNAs induce H3K27ac enrichment at target enhancers and put them into an active state conducive to gene activation. Notably, similar to NamiRNAs, Human Identical Sequences as exogenous small RNAs as critical pathogenic factors for RNA viruses, facilitate infectious disease progression. Collectively, this review systematically elucidates the theory of NamiRNA‐Enhancer‐mediated Gene Activation in combination with corresponding evidence, summarizes the functions and challenges of NamiRNAs, and discusses their potential in fundamental research and clinical applications.

## Graphical abstract

MiRNAs (miRNAs) regulate diverse biological processes across species. Unlike canonical miRNA‐mediated gene repression, Nuclear Activating miRNAs (NamiRNAs) can efficiently upregulate gene expression by targeting enhancers. This review systematically summarizes recent advances in NamiRNA research, elucidates the theory of NamiRNA‐Enhancer‐mediated Gene Activation, and discusses the challenges and applications of NamiRNAs in both fundamental research and clinical translation.

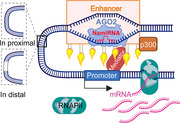

## 1 Introduction

Almost all living organisms are composed of cells, and specific gene expression patterns determine their identity and fate. As indispensable regulators of gene expression, microRNAs (miRNAs) are a class of small non‐coding RNAs ≈22 nucleotides (nt) in length^[^ ^1^ ^]^ and ubiquitously present in various species, including animals,^[^ ^2^ ^]^ plants,^[^ ^3^ ^]^ and viruses.^[^ ^4^ ^]^ Accumulating evidence underscores the critical role of miRNAs in individual development and disease. For example, deletion of miR‐1‐2 directly results in embryonic lethality with cardiac defects in mice.^[^ ^5^ ^]^ Moreover, knockout of miR‐122 leads to hepatocellular carcinoma in mice by 11 months of age.^[^ ^6^ ^]^ In fact, dysregulation of miRNAs often contributes to abnormal gene expression and promotes the progression of various diseases including tumors,^[^ ^7^ ^]^ neurological disorders,^[^ ^8^ ^]^ and metabolic diseases.^[^ ^9^ ^]^ As a result, miRNAs are considered as promising biomarkers and potential therapeutic targets for the diagnosis and treatment of diverse diseases.

Recently, Victor Ambros and Gary Ruvkun were jointly awarded the Nobel Prize in Physiology or Medicine for their groundbreaking contribution to the discovery of miRNAs and their post‐transcriptional gene regulation.^[^ ^10^ ^]^ It is widely acknowledged that miRNAs facilitate mRNA degradation or inhibit their translation by directly binding to their 3′ untranslated regions (3′ UTRs).^[^ ^11^ ^]^ Beyond these classical pathways, some studies have revealed that miRNAs can upregulate gene expression in the transcriptional level. For instance, miR‐373 significantly increases the mRNA levels of E‐cadherin via binding to its promoter.^[^ ^12^ ^]^ In addition, miR‐1 unexpectedly promotes the translation of specific mitochondrial transcripts through specific miRNA:mRNA base‐pairing.^[^ ^13^ ^]^ However, it remains challenging to derive general regulatory principles of miRNA‐mediated gene activation from these insightful studies.

Intriguingly, we identified a series of miRNAs in the nucleus that activate gene transcription by targeting enhancers through specific base‐pairing rules^[^ ^14^, ^15^, ^16^, ^17^, ^18^ ^]^ and termed them Nuclear Activating miRNAs (NamiRNAs). A representative example is miR‐24, which interacts with its target enhancer to significantly stimulate *FABP1* transcription.^[^ ^14^ ^]^ Further bioinformatic analysis revealed that over 75% of miRNAs in the human genome are located within enhancer regions,^[^ ^19^ ^]^ highlighting their roles in transcriptional gene activation. Consistent with our findings, Phillip A. Sharp and colleagues also found that multiple miRNAs associated with cancer hallmarks are marked by enhancers.^[^ ^20^ ^]^ Excitingly, based on the NamiRNA‐enhancer network, we discovered numerous novel pathogenic elements in RNA viruses and named them as Human Identical Sequences (HIS),^[^ ^21^ ^]^ further supporting the universality of NamiRNA‐mediated gene activation. In this review, we systematically summarize the recent advances in NamiRNA research and deeply discuss their potential applications in basic research and clinical practice.

## 2 MiRNAs from Non‐Canonical to Canonical

Currently, the roles and regulatory mechanisms of miRNAs have been extensively investigated in various physiological and pathological processes during development and disease. Based on existing evidence, we provide a detailed overview of the discovery and biogenesis of miRNAs, as well as their regulatory function involved in gene silencing.

### 2.1 A Breakthrough Discovery of miRNAs and Their Exponential Progress in Science

The first miRNA, lin‐4, was discovered by Ambros and Ruvkun in *Caenorhabditis elegans* (*C. elegans*) in 1993.^[^ ^22^, ^23^ ^]^ At that time, Ambros and Ruvkun unexpectedly found that the 3′ UTR of *lin‐14* mRNA contained multiple binding sites for lin‐4 during their investigation of the *C. elegans* development. They demonstrated that lin‐4 could inhibit the translation of lin‐14 by binding to its 3′ UTR.^[^ ^22^, ^23^ ^]^ However, as a non‐mainstream finding, it was difficult for scientists at the time to accept this novel gene regulatory pattern mediated by a small RNA, and no other groups followed up on their research. It was not until 2000 that Ruvkun et al. identified *let‐7* as a second miRNA in *C. elegans*, which inhibited gene expression by binding complementarily to its 3′ UTR.^[^ ^24^ ^]^ Notably, *let‐7* is detected in various animal species, including vertebrates and arthropods,^[^ ^25^ ^]^ implying that miRNAs may be conserved regulators of gene silencing via base‐pairing rules. Subsequently, an increasing number of studies have been published to investigate the biogenesis of miRNAs,^[^ ^26^, ^27^, ^28^ ^]^ their roles, and gene regulatory mechanisms during development and disease.^[^ ^29^, ^30^, ^31^ ^]^ Therefore, the discovery of miRNAs has ushered in an entirely new era for RNA regulation and provides us novel insights on organismal development and function.

### 2.2 Canonical Biogenesis Pathways of miRNAs

MiRNAs are encoded in the genome either as individuals or clusters. It has been reported that clustered miRNAs are often transcribed together as a single polycistronic transcript, which is subsequently processed into multiple mature miRNAs.^[^ ^32^ ^]^ The biogenesis of miRNAs involves several key steps, including the transcription of primary miRNAs (pri‐miRNAs), processing and transport of precursor miRNAs (pre‐miRNAs), and the formation of the miRNA‐induced silencing complex (miRISC). In the canonical pathway, pri‐miRNAs are transcribed in the nucleus by RNA polymerase II (Pol II), bearing a 5′ cap and a 3′ poly(A) tail.^[^ ^33^ ^]^ These pri‐miRNAs are then processed into ≈70 nucleotide pre‐miRNAs with a 2 nt 3′ overhang by the Microprocessor complex,^[^ ^34^ ^]^ which consists of DGCR8, Drosha, and other auxiliary factors. With the assistance of the nuclear export receptor Exportin‐5,^[^ ^35^ ^]^ pre‐miRNAs are exported to the cytoplasm. In the cytoplasm, pre‐miRNAs are cleaved at their terminal loops by Dicer, generating ≈20 nt miRNA:miRNA* duplex intermediates.^[^ ^36^ ^]^ One strand of the duplex, known as the guide strand, is selectively incorporated into the AGO protein to form the miRISC, while the passenger strand (miRNA*) is usually degraded.^[^ ^37^ ^]^ In addition to the canonical pathway, several non‐canonical miRNA biogenesis pathways have been identified, including Drosha‐independent and Dicer‐independent mechanisms,^[^ ^38^, ^39^ ^]^ suggesting that further studies should be carried out to explore the miRNA biogenesis in the future.

### 2.3 MiRNA‐Mediated Gene Silencing by Targeting 3′ UTRs

It is well known that miRNAs suppress gene expression through degrading the mRNAs or inhibiting their translation. Specifically, the “seed” sequences, spanning nucleotides 2nd to 8th of the miRNAs, are primarily responsible for recognizing target sites on mRNAs.^[^ ^40^ ^]^ In animals, due to their relatively low complementarity, miRNAs binding to the 3′ UTR of mRNAs typically suppress protein synthesis rather than induce mRNA degradation^[^ ^26^ ^]^ Specially, AGO proteins in miRISC can interact with translation initiation factors (such as eIF4E and eIF4G) and further decrease the binding of mRNA to ribosomes, consequently inhibiting translation initiation. Additionally, miRNAs in miRISC can recruit deadenylases to remove poly (A) tails from mRNAs, thereby reducing their stability and translation efficiency. Notably, miRNA‐mRNA binding can induce premature ribosome dissociation during translation, resulting in truncated polypeptide chains. Unlike these conventional mechanisms, miRNAs can also induce mRNA degradation when they exhibit high complementarity (particularly in coding regions), relying on the endonuclease activity of AGO proteins within miRISC.^[^ ^41^ ^]^ Therefore, the interaction between miRNAs and their target mRNAs represents a promising approach for gene silencing and warrants further exploration for clinical applications.

## 3 Current Dilemma of miRNA Research

In the in‐depth exploration of miRNA inhibition theory and its research challenges, we inevitably encounter a series of intricate and highly challenging issues. These problems resemble elaborately constructed mazes, significantly impeding our comprehensive and profound understanding of the miRNA world.

### 3.1 Neglecting miRNA‐Upregulated Genes Constitutes A “Cherry‐Picking” Bias

There has long been a research bias in miRNA studies, favoring “emphasizing downregulation over upregulation” in the context of gene expression regulation. Traditional research paradigms have mostly focused on the phenomenon where miRNAs bind to the 3′ UTRs of their target mRNAs, thereby suppressing gene expression. However, the regulatory function of miRNAs in gene upregulation has been labeled as indirect effects and received insufficient attention and remains poorly elucidated. This 1D mode of thinking undoubtedly limits our understanding of the full landscape of miRNA functions and overlooks the multiple roles that miRNAs may play within complex biological networks, resulting in cherry‐picking selective data reporting.

### 3.2 What Determines the Differential Functions of Sequence‐similar miRNAs?

The functional diversity among miRNA family members presents another significant research challenge. Despite sharing highly similar sequences, particularly in their seed sequences that determine the specificity of target gene recognition, these miRNAs exhibit vastly different biological functions in vivo. For example, the miR‐125 family (comprising miR‐125a and miR‐125b) includes three human homologs: hsa‐miR‐125a, hsa‐miR‐125b‐1, and hsa‐miR‐125b‐2, all of which share the same seed sequence.^[^ ^42^ ^]^ Intriguingly, miR‐125a promotes cell proliferation and migration in colon cancer,^[^ ^43^ ^]^ but miR‐125b downregulates the expression of *STAT3*, thereby inhibiting proliferation and inducing apoptosis of SW480 colon cancer cells.^[^ ^44^ ^]^ This “similar sequences but divergent functions” phenomenon not only complicates research but also challenges traditional functional prediction strategies reliant on sequence similarity. It prompts us to rethink: beyond the seed sequence, what other factors determine miRNA function?

### 3.3 Understanding the Functional Differences of miR‐Twins or miR‐Triplets

The miRNA twins (miR‐twins) or triplets (miR‐triplets) clearly denote the miRNAs with identical mature sequences but derived from distinct precursors located in various genomic loci (e.g., miR‐199a‐1 and miR‐199a‐2), which further deepens our confusion about miRNA functional specificity. Although these miRNAs share the same mature sequences originating from the different pri‐miRNAs, they exhibit markedly distinct biological function. For instance, hsa‐let‐7a‐1, hsa‐let‐7a‐2, and hsa‐let‐7a‐3 yield identical mature sequences despite being transcribed from different chromosomal loci. Notably, among this miR‐triplet, only let‐7a‐3 possesses a CpG island in its promoter region and shows downregulated expression in diabetic nephropathy.^[^ ^45^ ^]^ Such functional divergence may arise from their differential genomic sequences or positions of pri‐miRNAs. However, precise mechanistic elucidation will require additional experimental validation and theoretical support.

### 3.4 Differential Biological Function of miRNA in Cytoplasm and Nucleus

When we expand our research horizons to include nuclear‐localized miRNAs, an entirely new research dimension quietly opens up. A striking example is miR‐24, which exhibits opposing functional roles depending on its subcellular localization: while inhibiting gene expression in the cytoplasm, it plays a gene activation within the nucleus. Specially, NamiRNA miR‐24‐1 reactivates *FBP1* expression to suppress the Warburg effect in renal cell carcinoma cells, consequently inhibiting their proliferation and metastasis.^[^ ^46^ ^]^ In contrast, miR‐24‐1‐5p significantly reduces SHOX2 level and suppresses malignant phenotypes of clear cell RCC (ccRCC) cells in the cytoplasm.^[^ ^47^ ^]^ This phenomenon of “bidirectional functions of one miRNA” not only challenges our conventional understanding of miRNA function but also highlights the unique regulatory potential of nuclear activating miRNAs, namely NamiRNAs. NamiRNAs' localization and functional characteristics within the nucleus provide novel perspectives and experimental bases for understanding the multifaceted roles of miRNAs in gene regulation.

## 4 Non‐classical Phenomena Other Than miRNA‐Mediated Gene Silencing

To address the above dilemma regarding miRNAs, several recent works have been performed to explore the regulatory roles of miRNAs in gene activation at both transcriptional and post‐transcriptional levels. In this section, we classify these non‐canonical pathways of gene upregulation by miRNAs and observe that most of them are applicable only to a limited number of miRNAs in detail. Accordingly, there is an urgent need to build a systematic theory for miRNA‐mediated gene upregulation, which should be appropriate for a broad spectrum of miRNAs and easily verifiable across different disease contexts and developmental models.

### 4.1 Promoting Gene Transcription

#### 4.1.1 Interaction with Promoters

**Figure 1:**
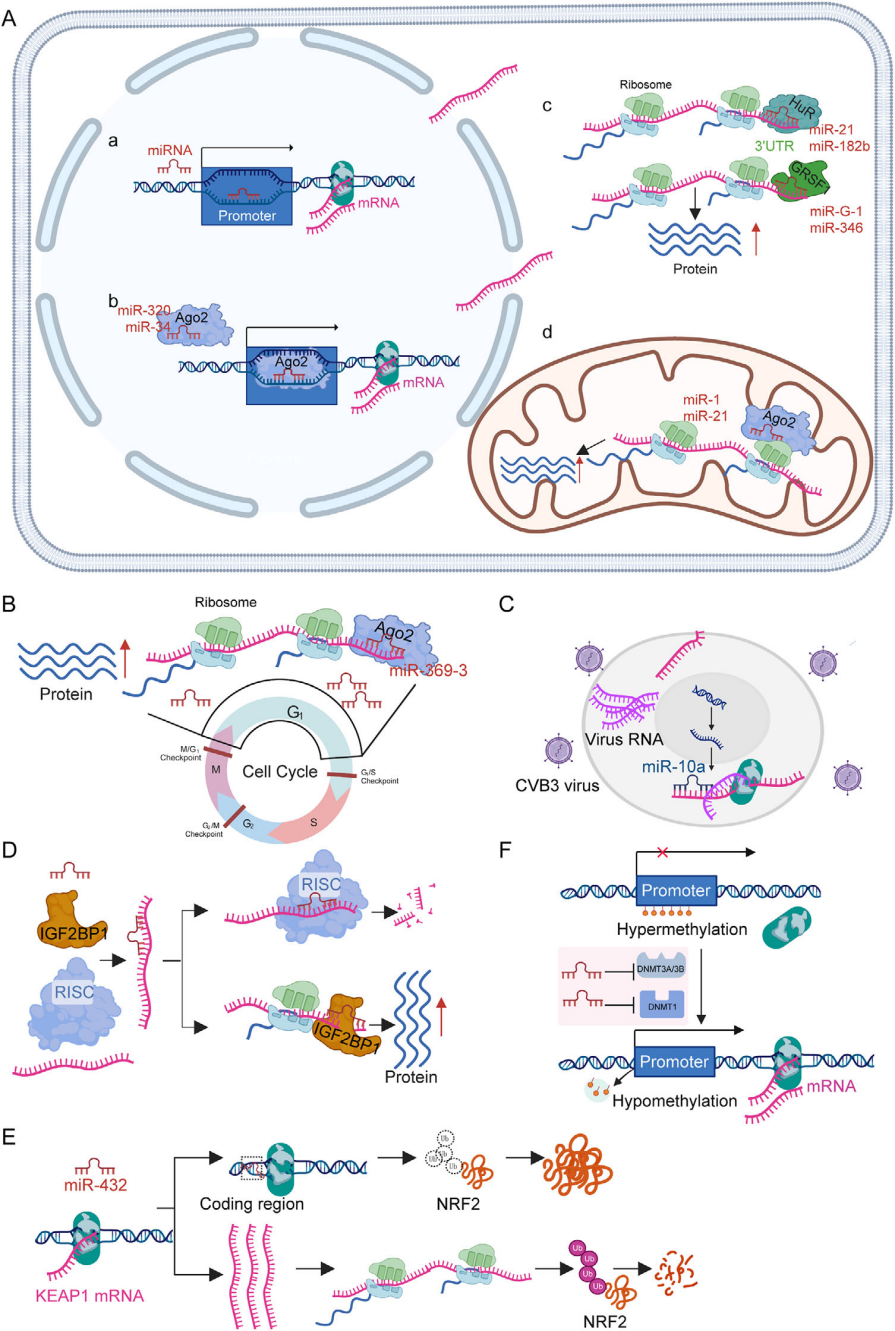
Non‐classical pathways of miRNA‐mediated gene upregulation (created with permission by BioRender). A) miRNAs upregulate gene expression at transcriptional and translational levels in different subcellular locations. In the nucleus, miRNAs promote transcriptional activation via binding to promoters (a). Besides, Ago2 with some miRNAs such as miR‐320 and miR‐34 can recruit RNA Poly II to enhance transcription (b). In the cytoplasm, some miRNAs stabilize target mRNAs and promote their translation (c). Other miRNAs, including miR‐1 and miR‐21, associate with ribosomes to facilitate mRNA translation in mitochondria (d). B) MiRNAs facilitate translation dependent on the phase of the cell cycle. During G1 phase, miR‐369 recruits AGO proteins to enhance translation, whereas this activity is suppressed in quiescent cells. C) Gene upregulation by miRNAs is in a sequence‐dependent rule. Host miR‐10a binds complementary sequences in the CVB3 viral genome, augmenting viral RNA and capsid protein production. D) MiRNA promotes translation by releasing mRNAs from miRISC dependent on IGF2BP1. E) MiRNAs indirectly upregulate gene expression by suppressing DNA methylation at promoters. F) MiRNAs indirectly increase gene expression via maintaining protein stabilization.

Although miRNAs are traditionally defined as post‐transcriptional repressors, recent studies have uncovered an unexpected role for certain miRNAs in promoting gene expression through direct interaction with gene promoters (**Figure** **1**A). This non‐canonical mode of action positions miRNAs as upstream regulators of transcription and chromatin state. For instance, miR‐205 binds to complementary sequences within the *PTEN* promoter, facilitating the recruitment of RNA Poly II and the histone acetyltransferase P300 to promote transcription in prostate cancer cells.^[^ ^48^ ^]^ Similarly, miR‐373 induces E‐cadherin and *CSDC2* expression by targeting their promoters,^[^ ^12^ ^]^ while miR‐324‐3p enhances *RelA* transcription, thereby linking promoter‐directed miRNA activity to NF‐κB signaling.^[^ ^49^ ^]^ Additionally, miR‐589 targets the *COX‐2* promoter to upregulate its transcription and modulate inflammatory pathways.^[^ ^50^ ^]^ Notably, the diversity of miRNA‐promoter interactions could take opposite biological functions. For example, upregulation of tumor suppressors such as *PTEN* by miR‐205 supports its antitumor role, whereas upregulation of Cyclin B1 induced by certain miRNAs has been implicated in tumor progression.^[^ ^51^ ^]^ These findings reveal a sequence‐specific mechanism by which miRNAs can function as direct transcriptional activators.

#### 4.1.2 Sequence‐Dependent Effects and RNA Polymerase Engagement

It is reported that miRNAs can function as transcriptional co‐activators, operating through both sequence‐specific interactions and direct engagement with the transcriptional machinery (Figure 1A). These nuclear miRNAs challenge the conventional cytoplasmic paradigm by regulating gene expression at the transcriptional level. A compelling example of sequence‐dependent effects is provided by miR‐10a*, which binds to the coding region of the coxsackievirus B3 genome to enhance viral RNA synthesis. This interaction recruits host transcriptional components independently of classical promoter elements, effectively acting as a *cis*‐acting enhancer of viral gene expression.^[^ ^52^ ^]^ Concurrently, nuclear miRNAs can directly interact with RNA polymerase II to modulate host gene transcription. For instance, in diabetic cardiomyopathy, nuclear miR‐320 promotes the expression of genes involved in fatty acid metabolism (e.g., *CPT1A*) by binding to Pol II at their promoters.^[^ ^53^ ^]^ A unifying theme across these studies is the role of miRNAs in resolving transcriptional pausing. For example, nuclear miRNAs have been shown to recruit the DDX21‐CDK9 complex to release paused Pol II, thereby promoting transcriptional elongation. Notably, this process, which is independent of miRNA sequence complementarity to DNA, highlights a *trans*‐acting mechanism where miRNAs act as scaffolds to recruit elongation factors, dynamically reshaping the transcriptional landscape.^[^ ^54^ ^]^ Collectively, these findings support a model in which miRNAs serve as dynamic transcriptional co‐regulators, finely tuning gene expression through both RNA‐DNA and RNA‐protein interactions in diverse physiological and pathological contexts.

### 4.2 Enhancing mRNA Translation

Recent studies have revealed that miRNAs, traditionally recognized as translational repressors, can paradoxically enhance their translation. These novel functions depend on interactions with RNA‐binding proteins (RBPs), sequence‐specific dynamics, mitochondrion localization, and cell cycle‐dependent regulation, illustrating a complex regulatory landscape.

In the context of RBPs as central mediators, GRSF1 and HuR exemplify RBPs that cooperate with miRNAs to activate translation (Figure 1A). For example, in cervical cancer, GRSF1 binds to MIR‐G‐1 to enhance the expression of TMED5 and LMNB1, driving nuclear autophagy and malignancy by recruiting translation machinery to target mRNAs.^[^ ^55^ ^]^ Similarly, HuR facilitates the miRNA‐mediated upregulation of NFI‐A in myeloid‐derived suppressor cells (MDSCs) during sepsis, potentially by stabilizing miRNA‐mRNA complexes or enhancing ribosome loading.^[^ ^56^ ^]^ Notably, GRSF1 also contributes to the function of miR‐346, which upregulates AGO2 to amplify the activity of other miRNAs, establishing a feed‐forward loop that exacerbates oncogenicity.^[^ ^57^ ^]^ These findings position RBPs as versatile scaffolds that redirect miRNAs from repression to activation in disease contexts. Regarding sequence‐dependent increase, the functional outcome of miRNA‐mRNA interactions can vary based on the extent of base pairing. Partial complementarity, particularly in the 3′ UTRs, allows miRNAs such as miR‐3 to relieve structural hindrances or recruit activating factors, thereby transitioning miRNAs from repressors to translational enhancers.^[^ ^58^ ^]^ In addition, miR‐10a binds to the 5′ UTRs of ribosomal protein mRNAs via imperfect complementarity, stimulating their translation to promote cell growth.^[^ ^58^ ^]^ This contrasts with classical seed‐region pairing, highlighting how sequence context dictates functional polarity. In terms of subcellular compartmentalization, mitochondrial miRNA activity reveals a GW182‐independent mechanism (Figure 1A). During muscle differentiation and in hypertensive rats, miR‐1 and miR‐21 with AGO2 enhance mitochondrial translation by directly interacting with mitochondrial ribosomes, thereby bypassing cytoplasmic GW182‐mediated repression.^[^ ^59^ ^]^ This suggests that miRNA function is rewired in organelles lacking core miRISC components, highlighting the subcellular environment as a critical determinant of miRNA activity.

Regarding cell cycle and temporal regulation, the translational enhancement by miRNAs is dynamically regulated throughout the cell cycle (Figure 1B). For instance, specific miRNAs enhance the translation of cell cycle‐promoting mRNAs (e.g., cyclins) during the G1‐S transition by displacing repressive complexes or recruiting initiation factors, thereby coupling translational control to cellular proliferation.^[^ ^60^, ^61^ ^]^ This temporal specificity implies that the cellular state and signaling cues dictate miRNA functionality. Furthermore, the cross‐talk between miRNAs and translational machinery is exemplified by the hepatitis C virus (HCV) RNA, which hijacks miR‐122. This miRNA binds to the 5′ UTR of HCV RNA to stabilize the viral genome and recruit ribosomes, illustrating how miRNAs can function as viral translation enhancers.^[^ ^62^ ^]^ Similarly, ribosomal protein mRNAs are directly increased by miR‐10a through 5′ UTR interactions (Figure 1C),^[^ ^63^ ^]^ suggesting that miRNAs may broadly fine‐tune ribosome biogenesis under stress or growth conditions.

### 4.3 Gene Expression Enhancement by Silencing the Silencer

Numerous studies have demonstrated that miRNAs can upregulate gene expression through various indirect roles, challenging the traditional perception of miRNAs as mere post‐transcriptional suppressors. These mechanisms encompass post‐transcriptional regulation, protein stability regulation, epigenetic modification, and genome‐level alterations, thereby revealing the intricate regulatory network of miRNAs in disease contexts.

The regulation of mRNA release and stability mediated by the miRISC complex involves IGF2BP1 (Figure 1D), which functions as an onco‐embryonic RNA‐binding protein that activates oncogenes in cancer. In particular, IGF2BP1 binds to and stabilizes E2F‐driven oncogenic mRNAs (e.g., *MYC*, *CCND1*), indirectly releasing these transcripts and enhancing their translation by competitively counteracting the inhibitory effect of the miRISC complex on mRNAs.^[^ ^64^ ^]^ This regulatory model, in contrast to the classical miRISC silencing function, underscores the capacity of RBPs to indirectly activate oncogenes by disrupting the miRNA pathway, thus providing a theoretical foundation for the development of drugs targeting RBPs, such as small molecule inhibitors. Moreover, miRNA can indirectly enhance the stability of specific proteins by inhibiting negative regulators of target proteins (Figure 1E). For instance, miR‐432 diminishes the ubiquitination and subsequent degradation of NRF2 by directly inhibiting KEAP1, a NRF2's E3 ubiquitin ligase, thereby activating the antioxidant pathway.^[^ ^65^ ^]^ This indirect effect of miR‐432 on ubiquitination expands the functional scope of miRNAs and may represent a therapeutic target in cancers associated with oxidative stress.

Epigenetic activation driven by DNA demethylation occurs when members of the miR‐29 family, such as miR‐29b, induce genome‐wide DNA hypomethylation (Figure 1F). This process is facilitated by the direct inhibition of DNA methyltransferases (DNMT3A/3B) and DNMT1, leading to the reactivation of tumor suppressor genes, including *CDKN2B* and *ESR1*. This mechanism plays a significant role in suppressing cancer in acute myeloid leukemia.^[^ ^66^ ^]^ Similarly, retinoic acid‐induced miRNAs promote cell maturation during neuroblastoma differentiation by inhibiting DNMTs, which triggers the demethylation of neural gene promoters, such as *NGFR*.^[^ ^67^ ^]^ Furthermore, miR‐29b can upregulate the fetal hemoglobin gene through the same mechanism, offering a novel strategy for the epigenetic therapy of β‐thalassemia.^[^ ^68^ ^]^ Collectively, these studies suggest that miRNAs can indirectly reshape chromatin states and upregulate silenced genes by regulating epigenetic modification enzymes. Oncogene enhancement resulting from miRNA gene deletion can relieve the inhibition of downstream targeted genes, thereby indirectly driving carcinogenic pathways. For instance, the genomic deletion of miR‐101 leads to the overexpression of the histone methyltransferase EZH2, which promotes cancer metastasis by silencing tumor suppressor genes, such as *CDH1*, through H3K27me3 modification.^[^ ^69^ ^]^ This indirect function of miR‐101 on H3K27me3 underscores the role of miRNAs as “guardians” of the genome, whose deletion can indirectly activate cancer‐promoting signaling via epigenetic reprogramming.

Together, these studies illustrate the complexity of miRNAs as multilevel regulatory hubs. Their indirect upregulation roles provide new insights into the treatment of cancer, blood diseases, and differentiation disorders. Future research should integrate multi‐omics and single‐cell techniques to analyze the precise roles of miRNA‐mediated indirect regulatory networks in spatiotemporal dynamics.

## 5 NamiRNAs and Enhancers: Keys to Resolving Classical miRNA Dilemmas

To build a systematic theory for miRNA‐mediated gene transcription, we focused on miRNAs and enhancers and observed that both of them show strong tissue‐specific and cell‐specific characteristics. Subsequently, we designed a series of experiments and revealed a general phenomenon that miRNAs located in genomic enhancer regions significantly activate specific genes in the corresponding cells. Based on these findings, we first proposed a novel theory of NamiRNA‐Enhancer‐mediated Gene Activation that NamiRNAs epigenetically activate gene transcription by interacting with target enhancers (A detailed discussion is presented in Section 8), which provides an excellent model for gene activation by miRNAs and serves as a key solution for the classic miRNA's dilemma.

### 5.1 Tissue‐Specific Enhancers

Enhancers are critical *cis*‐regulatory elements that, although non‐coding themselves, can significantly boost the transcriptional activity of target genes upon binding by specific transcription factors. A defining feature of enhancers is their spatiotemporal specificity; their activity is tightly dependent on the cellular context, including tissue type, developmental stage, and the availability of relevant transcription factors. These special positional enhancers, functioning as key regulatory “commanders”, determine whether, when, and where a gene is expressed.

The tissue‐specific activity of enhancers plays a decisive role in fundamental biological processes such as sex determination. Although the Y chromosome‐encoded *SRY* gene has long been considered the trigger for male development, it is now recognized that SOX9 and its upstream enhancers (eSR‐A, eSR‐B, and eALDI) are the true arbiters of sex fate. eSR‐A and eSR‐B directly activate *SOX9* transcription, whereas eALDI promotes *SRY* expression, indirectly initiating SOX9 activation.^[^ ^70^ ^]^ Functional defects in any of these enhancers can impair SOX9 expression, leading to disorders of sex development, even in individuals possessing a Y chromosome.

Similarly, the expression of Sonic hedgehog (*Shh*) during limb development depends on a distant, tissue‐specific enhancer known as Zone of Polarizing Activity Regulatory Sequence (ZRS).^[^ ^71^ ^]^ ZRS is exclusively active during the limb bud stage and controls the spatial initiation of *Shh* expression. During snake evolution, basal species like pythons retain a relatively conserved ZRS sequence, allowing residual limb expression, whereas advanced snakes such as cobras exhibit highly mutated ZRS regions, resulting in the complete loss of limb developmental potential. These findings highlight the crucial role of tissue‐specific enhancers in shaping morphological characteristics.

### 5.2 Tissue‐Specific miRNAs

Analogous to enhancers, miRNAs display highly tissue‐ and cell‐type‐specific expression patterns. For instance, miR‐1 is predominantly expressed in muscle tissues,^[^ ^72^ ^]^ its deletion in *Drosophila* leads to impaired muscle differentiation and lethality,^[^ ^73^ ^]^ while deletion of *miR‐1‐2* in mice causes cardiac developmental defects resulting in lethality in approximately half of the embryos.^[^ ^5^ ^]^ Another example is miR‐122, which is highly enriched in the liver, accounting for ≈ 70% of total hepatic miRNAs, and is crucial for the replication of the hepatitis C virus.^[^ ^74^ ^]^

Despite accumulating evidence for the tissue specificity of miRNAs, the mechanisms underlying such selective expression remain incompletely understood. Emerging studies suggest that the chromatin state and upstream enhancers of miRNA genes may contribute to their expression patterns. These insights imply that enhancers and miRNAs may function in a coordinated regulatory network, rather than acting independently, to govern tissue‐specific gene regulation.

Recent evidence has revealed that miRNAs are not only post‐transcriptional regulators but also closely associated with *cis*‐regulatory elements such as enhancers in terms of their genomic localization and expression control.^[^ ^20^ ^]^ Notably, the research group led by Phillip A. Sharp investigated the interplay between super‐enhancers (SEs) and miRNAs,^[^ ^20^ ^]^ discovering that the expression of several miRNAs is directly regulated by SEs. In mouse embryonic stem cells, for example, two stem cell‐specific miRNA clusters (*miR‐290‐295* and *miR‐106a‐363*) have been identified as SE‐associated miRNAs.^[^ ^75^, ^76^ ^]^ These miRNAs function through Ago2‐mediated post‐transcriptional repression mechanisms to regulate cell cycle progression and developmental processes.

### 5.3 Genomic Co‐Localization of miRNAs and Enhancers

**Figure 2:**
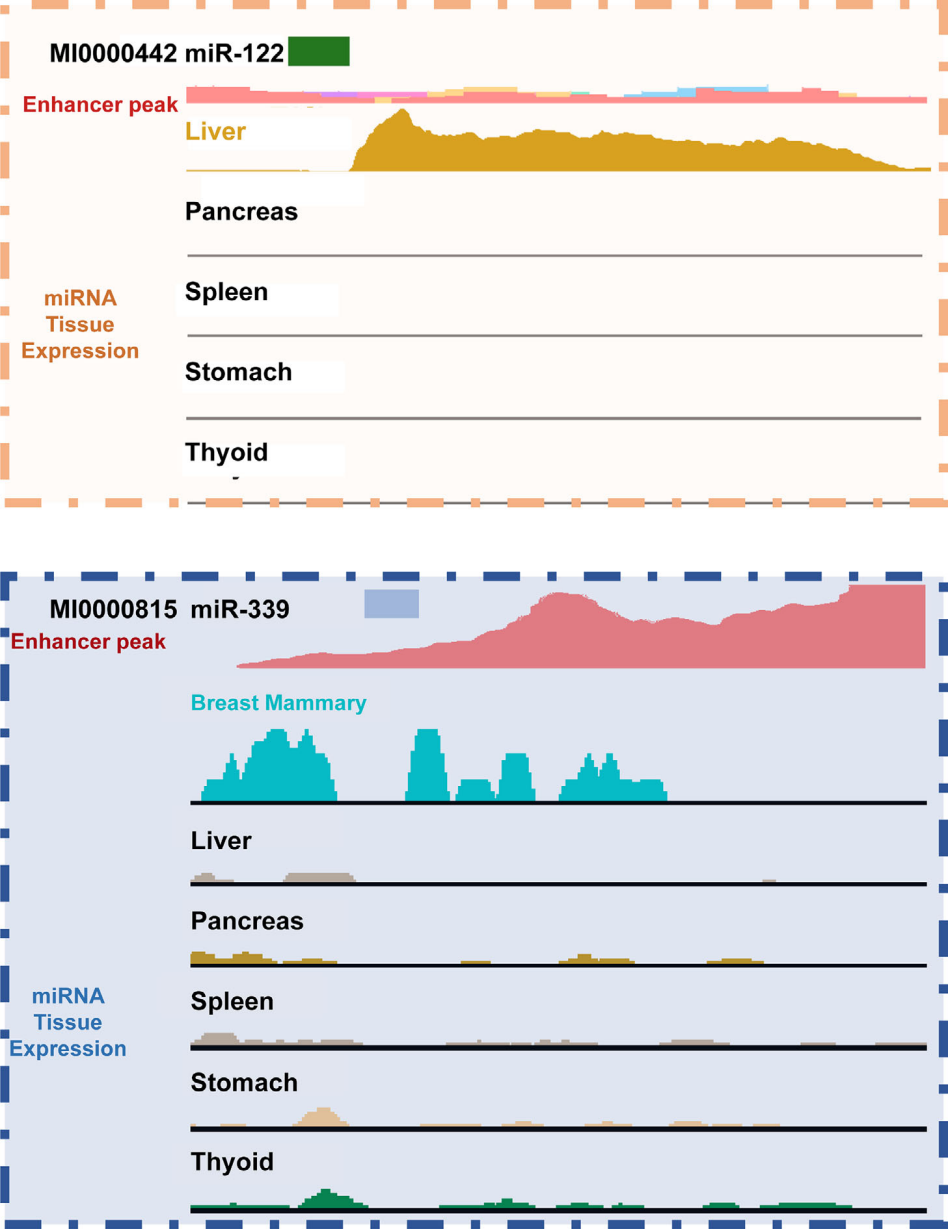
Tissue‐specific miRNAs are overlapped with enhancers. Enhancers are identified by H3K27ac peaks. The expression of miRNAs is quantified based on the RNA‐seq data. MiR‐122, which overlaps with an enhancer, exhibits liver‐specific expression but shows no significant enrichment in other tissues. Similarly, miR‐339 is localized within enhancer regions and shows high expression in mammary breast tissue.

In our systematic analysis of 1594 human miRNA precursors across multiple tissues and cell types, we found that ≈ 300 miRNAs are located within genomic regions enriched for canonical enhancer marks (H3K4me1 and H3K27ac).^[^ ^14^ ^]^ Most of these miRNAs are localized within the nucleus and possess the capacity to associate with enhancer elements and promote transcriptional activation of genes across the genome. As shown in **Figure** **2**, liver‐specific miR‐122 and breast mammary‐specific miR‐339 are overlapped with the peak of H3K27ac, indicating that tissue‐specific miRNAs are located in genomic enhancers and present tissue‐specific expression. Our results found that miR‐122 activates the expression of liver‐specific transcription factors. Importantly, this gene activation potential is dependent on the structural integrity of the enhancer; deletion of the enhancer regions encompassing these miRNAs significantly reduces their ability to upregulate target genes, thereby impairing tumor cell proliferation and migration.

These findings collectively highlight the overlapping genomic distribution of miRNAs and enhancers, revealing their cooperative roles in tissue‐specific gene regulation. Enhancers serve to promote gene transcription, while miRNAs themselves may also possess gene‐activating potential. This dual functionality underscores a complex and integrated regulatory mechanism in which enhancer‐driven miRNAs (including SE‐miRNAs) contribute not only to post‐transcriptional silencing but also to the spatial and temporal control of gene expression at multiple regulatory layers.

## 6 NamiRNAs Effectively Activate Gene Transcription

**Figure 3:**
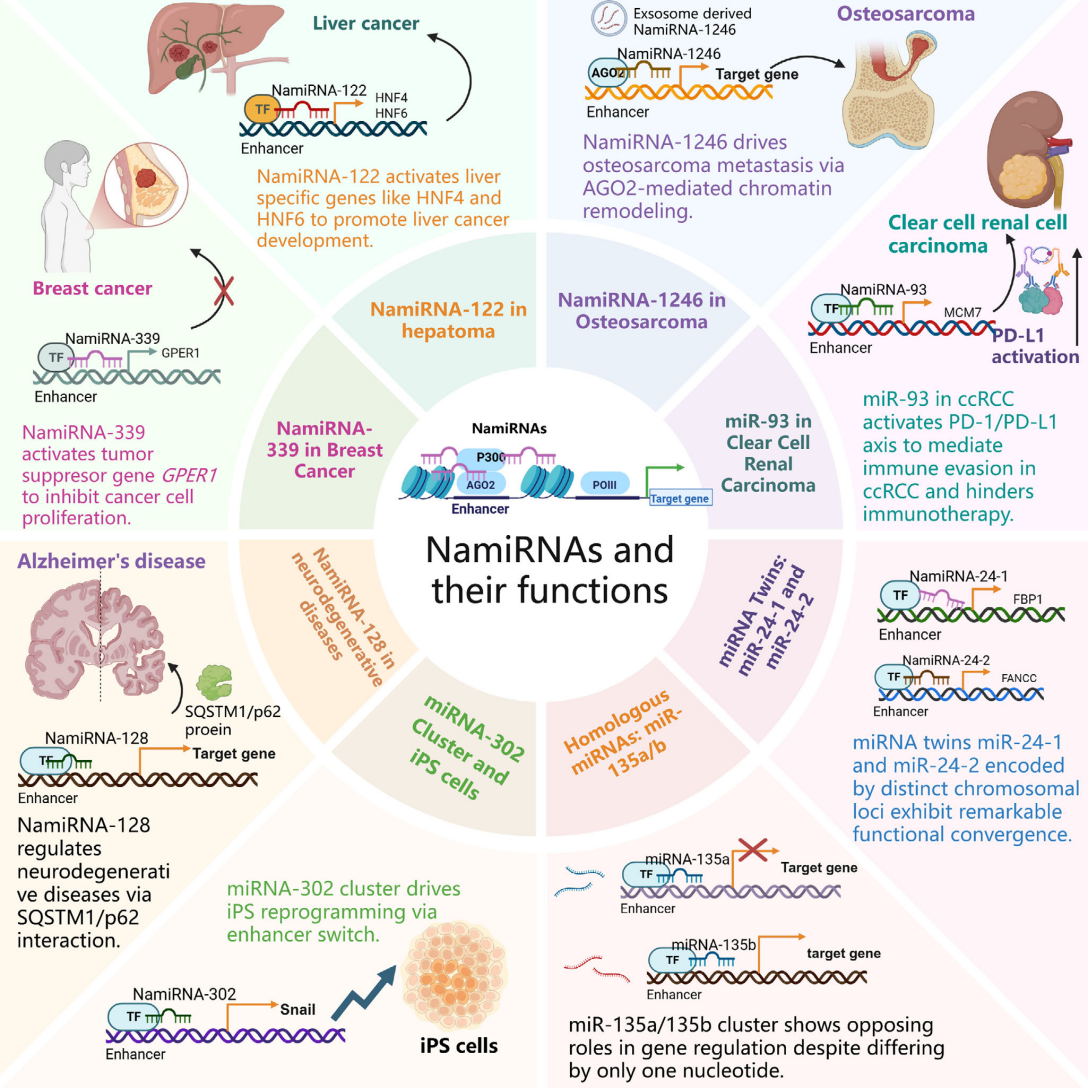
NamiRNA and their functions in different diseases (created with permission by BioRender). NamiRNAs play multifaceted roles in diseases. NamiRNA suppresses breast cancer proliferation by targeting the GPER1 enhancer. NamiRNA‐1246 drives osteosarcoma metastasis via AGO2‐mediated chromatin remodeling. NamiRNA‐93 fuels immunotherapy resistance in ccRCC by activating PD‐1/PD‐L1. NamiRNA‐24 twins act as both transcriptional activators and post‐transcriptional silencers. NamiRNA‐135a/b shows antagonism in NSCLC. NamiRNA‐128 is linked to neurodegenerative diseases.

NamiRNAs bridge transcriptional and post‐transcriptional control, playing multifaceted roles in tumorigenesis, metastasis, immune evasion, and neurodegenerative diseases (**Figure** **3**). Their ability to modulate oncogenic or tumor‐suppressive pathways via nuclear‐niche targeting positions makes them as critical regulators in cancer biology, offering novel avenues for precision therapeutics.

### 6.1 NamiR‐339 in Breast Cancer: Suppressing Proliferation via Targeting *GPER1* Enhancer

In breast cancer research, miR‐339 has been identified as a potent suppressor of cancer cell proliferation and growth.^[^ ^15^ ^]^ This miRNA exerts its tumor‐inhibitory effects by precisely targeting the enhancer region of the tumor suppressor gene *GPER1*, thereby upregulating its expression. Enhanced *GPER1* activity disrupts estrogen‐driven proliferative signaling and induces cell cycle arrest, effectively curbing tumor expansion. This discovery not only elucidates a novel epigenetic regulatory mechanism but also highlights miR‐339 as a promising candidate for targeted therapies in hormone receptor‐positive breast cancers.

### 6.2 NamiRNA‐1246 in Osteosarcoma: Promoting Metastatic Invasion Targeting *MMP1* Enhancer

Investigations into osteosarcoma have uncovered the pivotal role of NamiRNA‐1246 in driving tumor aggressiveness.^[^ ^18^ ^]^ This non‐coding RNA facilitates metastatic progression through AGO2‐mediated chromatin remodeling, which activates pro‐invasive genes such as *MMP1*. By orchestrating enhancer‐promoter looping, NamiRNA‐1246 rewires transcriptional programs to promote extracellular matrix degradation and mesenchymal transition. These findings deepen our understanding of osteosarcoma metastasis and underscore the therapeutic potential of disrupting NamiRNA‐AGO2 interactions to inhibit tumor dissemination.

### 6.3 MiR‐93 in Clear Cell Renal Carcinoma: Fueling Immunotherapy Resistance via PD‐1/PD‐L1 Activation

In clear cell renal cell carcinoma, miR‐93 emerges as a key mediator of immune evasion. It activates the PD‐1/PD‐L1 immune checkpoint axis by suppressing *PTEN*, which hyperactivates the PI3K/AKT pathway and upregulates PD‐L1 expression on tumor cells. This mechanism induces T cell exhaustion and cripples anti‐tumor immunity, rendering immunotherapies ineffective. Patients with high miR‐93 expression exhibit significantly reduced response rates to PD‐1 inhibitors, emphasizing its role as a biomarker and therapeutic target. Combining miRNA inhibitors with immune checkpoint blockade may overcome resistance and restore immune surveillance in ccRCC.

Generally, NamiRNAs exemplify the complexity of cancer regulation, operating at the intersection of epigenetics, transcriptional control, and immune modulation. Their tissue‐specific interactions with chromatin and protein complexes (e.g., AGO2) enable precise targeting of oncogenic networks, influencing tumor initiation, metastasis, and therapy resistance. The cases of miR‐339, NamiRNA‐1246, and miR‐93 illustrate how these molecules drive diverse pathological processes while offering actionable targets. Future therapies leveraging NamiRNA inhibition or mimicry, particularly in combination with existing modalities like immunotherapy, hold transformative potential for precision oncology. Unraveling their context‐dependent roles will be critical for developing tailored interventions across cancer types.

### 6.4 Reciprocal Regulation of miRNA Twins: miR‐24‐1 and miR‐24‐2

The miRNA twins miR‐24‐1 and miR‐24‐2, despite being encoded by distinct chromosomal loci (chr9q22.32 and chr19p13.12, respectively), exhibit remarkable functional convergence and unique regulatory features. Both miR‐24‐1 and miR‐24‐2 act as non‐canonical transcriptional activators by directly binding to enhancer regions. This interaction facilitates chromatin remodeling, enhances RNA polymerase II recruitment, and upregulates transcription of genes proximal to their binding sites. For example, miR‐24‐1 activates genes near its host locus (e.g., *C9orf50*), while miR‐24‐2 stimulates the expression of neighboring genes such as *C19orf44*, demonstrating locus‐specific enhancer activation.

In the nucleus, these miRNAs modulate transcriptional programs by stabilizing enhancer‐promoter looping, thereby amplifying oncogenic or developmental signals in a context‐dependent manner. Concurrently, they retain canonical miRNA activity in the cytoplasm, where their identical mature sequences (5′‐UGCCUAC‐3′) post‐transcriptionally silence target mRNAs through seed sequence pairing. Key targets include pro‐apoptotic genes (e.g., *BCL2L11*) and cell cycle regulators (e.g., *CDKN1B*), enabling bidirectional regulation of cellular processes.

The functional duality of miR‐24‐1/24‐2 challenges the traditional view of miRNAs as purely gene‐repressing molecules. Their ability to act as enhancer‐triggered transcriptional activators while maintaining cytoplasmic RNA interference highlights an evolutionary adaptation for fine‐tuned gene regulation. This dual functionality may explain their conserved roles in tissue development, cancer progression (e.g., ccRCC), and metabolic reprogramming, where precise spatiotemporal control of gene networks is critical. In summary, miR‐24‐1 and miR‐24‐2 exemplify a paradigm of miRNA twins that harmonize nuclear transcriptional activation with cytoplasmic post‐transcriptional silencing, enabling multilayered control of cellular homeostasis.

### 6.5 Divergent Regulation of Homologous miRNAs: miR‐135a and miR‐135b

The miR‐135a/135b cluster, a pair of highly homologous microRNA family differing by only a single nucleotide in their mature sequences, exhibits striking functional antagonism in non‐small cell lung cancer (NSCLC).^[^ ^16^ ^]^ Despite their near‐identical structures, these miRNAs demonstrate opposing expression patterns and biological roles, underscoring the complexity of miRNA‐mediated regulatory networks in oncogenesis.

In NSCLC, miR‐135a is frequently downregulated and functions as a critical suppressor of tumor progression. Through epigenetic reprogramming of oncogenes, miR‐135a attenuates cancer cell proliferation, migration, and invasive potential, positioning it as a candidate therapeutic target for restoring aberrant gene expression in NSCLC. Conversely, miR‐135b displays oncogenic upregulation in NSCLC tissues, where it actively promotes malignancy. Unlike its counterpart, miR‐135b engages distinct enhancer landscapes to enhance pro‐tumorigenic gene expression. This dualistic behavior highlights context‐dependent functional plasticity within homologous miRNA families.

The reciprocal activities of miR‐135a/135b exemplify how subtle sequence variations can drastically alter miRNA functionality. Their opposing roles in NSCLC pathogenesis suggest the potential for combinatorial therapeutic strategies: restoring miR‐135a levels while inhibiting miR‐135b could synergistically disrupt oncogenic circuits. This study illuminates the intricate interplay between homologous miRNAs in cancer biology, emphasizing that even minor sequence discrepancies can dictate divergent regulatory outcomes. Future investigations into the tissue‐specific and context‐dependent functions of miRNA families may uncover novel therapeutic avenues for NSCLC management.

### 6.6 The Third Practical Approach of iPS Cell Reprogramming: miRNA‐302 Cluster

Current studies have confirmed that stem cell‐specific miRNAs possess the biological capacity to induce somatic cell reprogramming. Our research further elucidates the critical regulatory role of the miRNA‐302 cluster (comprising mmu‐miR‐302a, 302b, 302c, 302d, and 367) located on mouse chromosome 4. Mechanistic investigations reveal that this cluster enhances the pluripotency maintenance of induced pluripotent stem (iPS) cells by activating transcription factors associated with epithelial‐mesenchymal transition, such as *Cdh1* and *Snail*. Experimental data demonstrated that the miRNA‐302 family not only regulates core transcriptional networks of pluripotency but also maintains the pluripotent state of iPS cells through epigenetic modification pathways that reshape chromatin accessibility.

Compared to traditional reprogramming methods, miRNA‐mediated processes exhibit unique biological characteristics: (1) Their mechanism operates independently of exogenous transcription factors (OSKM: OCT4/SOX2/KLF4/c‐MYC); (2) The miRNA regulatory system demonstrates superior target specificity and reduced genomic integration risks relative to chemical small‐molecule induction strategies. These findings suggest that miRNA‐based reprogramming technology may transcend the limitations of current iPS induction paradigms, establishing it as the third distinct reprogramming paradigm following transcription factor combinations (OSKM) and chemical approaches. This theoretical framework provides a critical foundation for developing genome integration‐free, high‐safety cell reprogramming technologies while offering new perspectives for deciphering the epigenetic regulatory networks underlying cell fate transitions.

### 6.7 NamiRNA‐128 and Neurodegenerative Diseases

NamiRNA‐128 has been found to potentially play a crucial role in the regulation of neurodegenerative diseases by interacting with the nuclear SQSTM1/p62 protein in our recent work. This interaction is pivotal in modulating the progression of Alzheimer's disease and other neurodegenerative conditions, thereby unveiling a novel function of NamiRNA beyond its established role in tumor regulation. The specific mechanism through which NamiRNA‐128 interacts with SQSTM1/p62 involves intricate molecular pathways that influence neuronal health and survival. By targeting these pathways, NamiRNA‐128 could potentially alter the course of neurodegeneration, offering a promising avenue for therapeutic intervention.

Furthermore, the discovery that NamiRNA‐128 is involved in neurodegenerative diseases suggests a broader role for NamiRNAs in neurological disorders. This opens up new possibilities for exploring the therapeutic potential of NamiRNAs in a wide range of neurological conditions, including Parkinson's disease, Huntington's disease, and amyotrophic lateral sclerosis.

### 6.8 Other Supporting Evidence for NamiRNA‐Mediated Gene Activation

Emerging evidence supports our findings that NamiRNA regulates gene expression by functioning as an enhancer triggers. In NSCLC, miR‐26a‐1 directly engages enhancer elements, increasing H3K27ac deposition and activating the transcription of tumor suppressor genes (TSGs) (such as *VILL*), thereby suppressing NSCLC cell proliferation and migration.^[^ ^17^ ^]^ Conversely, miR‐492 acts as an oncogenic NamiRNA, exhibiting marked overexpression in pancreatic cancer.^[^ ^77^ ^]^ In particular, miR‐492 transcriptionally upregulates neighboring genes (*NR2C1*, *NDUFA12*, and *TMCC3*), which converge on the TGF‐β/Smad3 pathway to drive the epithelial‐mesenchymal transition. Besides, the interaction between miRNAs and enhancers regulates immune response. For example, miR‐223 promotes PPARγ‐dependent alternative macrophage activation by stabilizing enhancer–promoter looping at PPARγ‐targeted loci, exemplifying direct miRNA‐enhancer crosstalk in immune regulation.^[^ ^78^ ^]^ Based on the NamiRNA‐enhancer network, it is convenient for us to define systematically the potential miRNAs that interact with the enhancer to activate transcription and explore their function in different diseases. These accumulated data highlight a broader epigenetic role for NamiRNAs beyond canonical miRNA silencing and opens new avenues for therapeutic intervention.

## 7 Unanticipated Breakthroughs Beyond NamiRNAs: Human Identical Sequences

Endogenous miRNAs play an important regulation role in enhancer activity and gene expression related to disease development, while exogenous miRNAs, such as small RNAs or miRNAs encoded by a virus, also interact closely with host enhancers.^[^ ^21^, ^79^ ^]^ We found that the SARS‐CoV‐2 has the same nucleotide sequence with the human genome, referred to as Human Identical Sequences (HISs), which can not only activate the expression of neighboring genes, but also activate the expression of distant genes by targeting host enhancers, which share similar gene activation mechanisms with NamiRNAs.^[^ ^21^ ^]^ Besides, we identified hepatitis B virus (HBV) Sequences Integrated To the Enhancer, termed as “HBV‐SITEs”. HBV‐SITEs were novel oncogenic elements by interacting with host enhancers to promote the hepatocellular carcinoma (HCC) development and progression, and small nucleotide drugs targeting HBV‐SITEs may provide an effective approach for the treatment and blockade of HCC progression.^[^ ^80^ ^]^

### 7.1 Discovery and Characteristics of Human Identical Sequences

Viruses can produce small RNAs, referred to as virus‐derived small RNAs (vsRNAs), which can affect the pathogenic processes of virus infection. For example, vsRNAs produced by the influenza A virus can promote viral RNA synthesis.^[^ ^81^ ^]^ VsRNA‐N derived from the N gene of SARS coronavirus (SARS‐CoV) leads to pulmonary inflammatory reaction.^[^ ^82^ ^]^ Therefore, vsRNAs are very important for viral infection.

SARS‐CoV‐2 infection began to spread around the world since 2019, which seriously threatened people's health and destroyed the global market economy. Building on over a decade of NamiRNA and the unique understanding of viral pathogenesis,^[^ ^14^ ^]^ we found that there are five nucleotide sequences in SARS‐CoV‐2 genome that are identical to segments of the human genome, each ranging from 24 to 27 nucleotides in length. Further analysis shows that more than 100 kinds of human pathogenic RNA viruses, including avian influenza virus and Zika virus, harbor short nucleotide sequences identical to those in the human genome.^[^ ^21^ ^]^ Finally, given that the short nucleotide sequences in these pathogenic RNA viruses are the same as the human genome, we termed these short RNA elements as Human Identical Sequences.

Subsequently, using a combination of cellular and molecular assays, we experimentally confirmed that HISs of SARS‐CoV‐2 can indeed upregulate the genes expression related to COVID‐19 progression by interacting with enhancers, which is consistent with the clinical indicators observed in COVID‐19 patients. Interestingly, some identical sequences were also found between the genomes of SARS‐CoV‐2 and its potential hosts, which were called Host Identical Sequences (HISs). However, the same sequences were not found in non‐COVID‐19 host chickens, indicating that the HISs of RNA viruses help trace the intermediate host of the virus or could be the key to the specificity of virus infection species.

Notably, these HISs exhibit the following characteristics.^[^ ^21^, ^79^ ^]^ First, HISs are ≥ 20 nucleotides in length, identical to the human genome. Second, the precursors of HISs in viruses can form miRNA‐like structures that may generate miRNA‐like small RNAs. Third, HIS fragments are located in enhancer regions of the human genome, which can potentially activate genes by targeting enhancers and promote the process of virus‐related diseases.

The discovery of HISs provides both theoretical foundations investigating the pathogenesis of diverse RNA viruses and novel perspectives for studying virus‐host interactions. Furthermore, it offers potential targets for antiviral drug development, potentially establishing new research frontiers in RNA virology and associated diseases. Antiviral therapeutics targeting viral nucleic acids are poised to emerge as a promising treatment paradigm, which may ultimately lead to successful clinical interventions against pathogenic RNA viruses.

### 7.2 HISs as Essential Pathogenic Factors of RNA Viruses

HISs of SARS‐CoV‐2 can promote COVID‐19 progression by inducing cytokine storm by activating inflammation‐related genes, and enhancing SARS‐CoV‐2 infection through upregulation of virus receptor *ACE2*. Importantly, HISs of SARS‐CoV‐2 can promote COVID‐19 progression by inducing hyaluronan accumulation through activating host enhancer enrichment and *HAS2* expression. In particular, the accumulation of hyaluronan directly contributes to lung lesions in severe COVID‐19 patients through excessive water molecule binding.^[^ ^21^, ^83^ ^]^ The research also found that these HISs could activate the expression of inflammation‐related genes in HEK293T, MRC5 and HUVEC cell lines, which indicated that SARS‐CoV‐2 could activate non‐immune cells to express inflammation‐related genes, eventually promoting the inflammatory reaction of human kidney, lung and vascular endothelium, which may be an important cause of multiple organ failure in COVID‐19. Therefore, HISs play a key pathogenic role in COVID‐19 through targeting the enhancer to activate gene transcription, mirroring the regulatory pattern of the NamiRNA‐enhancer network.

Notably, HISs activate gene expression in an AGO2‐dependent way.^[^ ^21^ ^]^ Following host cell infection, pathogenic RNA viruses (such as SARS‐CoV‐2) initiate replication while simultaneously producing various RNA transcripts. These transcripts can form HIS precursors with miRNA‐like hairpin structures, which are subsequently processed into mature HIS by relevant enzymes. With AGO2 facilitation, HISs interact with enhancers to induce the accumulation of H3K27ac and increases the activities of enhancers. This process ultimately promotes the transcription of both proximal and distal target genes through distinct protein complex mechanisms.

In addition, HISs from other viruses such as HIV, could also trigger T cell enhancer activity and upregulate gene expression related to AIDS progression. Based on the discovery of HISs, a viral nucleic acid pathogenic hypothesis was proposed: the same nucleotide fragments between the genomes of viruses and hosts may be the key elements for virus infection and diseases progression by targeting host cell enhancers.

### 7.3 HBV‐SITEs as Important Pathogenic Factors for HBV‐Induced Diseases

HBV DNA integration is the main contributor to HCC development.^[^ ^84^ ^]^ Recently, the HBV integrated non‐coding sequence itself has been found to act as a regulator for HCC development.^[^ ^80^ ^]^ Our study identified nine fragments of HBV Sequences Integrated To Enhancer, termed as “HBV‐SITEs”. Notably, HBV‐SITE‐1, which showed the highest integration frequency, served as a key pathogenic factor that upregulated the oncogene expression by modulating host enhancer activity, thereby promoting HCC proliferation and migration. In addition, the expression of HBV‐miR‐2 embedded in HBV‐SITE‐1 progressively increased in the plasma of HCC patients compared to those with CHB and liver cirrhosis, suggesting its potential as a biomarker for monitoring HCC progression. Furthermore, a single base change in HBV‐SITE‐1 of genotype H didn't significantly accelerate the tumorigenic phenotype, implying that this variation may reduce carcinogenicity of genotype H. Notably, targeting HBV‐SITEs downregulated tumor gene expression by reducing enhancer activity, and significantly inhibited tumor growth and HCC progression in animal models. Accordingly, targeting HBV‐SITEs could represent an effective therapeutic strategy to treat and block the HCC progression.

Therefore, HBV‐SITEs were identified as novel oncogenic elements for HCC, providing insightful perspectives for understanding other cancers caused by oncogenic DNA viruses. These findings support that viral nucleotide sequences are vital pathogenic substances beyond viral proteins. Targeting and modulating their enhancer activities may represent a novel therapeutic strategy for DNA viruses‐induced cancers.

### 7.4 Virus‐Derived miRNAs Act as Vital Disease Factors

More and more research proves that virus‐encoded miRNAs may be a common phenomenon. At present, it is believed that 20%–25% of human cancers are caused by virus infection, including liver cancer caused by persistent hepatitis B virus, cervical cancer caused by human papillomavirus (HPV), and nasopharyngeal cancer caused by Epstein‐Barr virus (EBV) infection.^[^ ^85^ ^]^ All three oncogenic viruses can encode virus‐derived miRNAs and play essential regulatory roles in tumorigenesis. For example, miRNAs encoded by EBV play a pivotal role in cell proliferation, differentiation, apoptosis, and tumorigenesis. EBV‐miR‐BART2‐5p can bind to the 3′ UTR of virus DNA polymerase BALF5, degrade BALF5, and maintain EBV in a latent infection state.^[^ ^86^ ^]^ EBV‐miR‐BART20‐5P maintains EBV in a latent infection state by acting on BZLF1.^[^ ^87^ ^]^ EBV‐miR‐BART1 down‐regulate *PTEN* to promote the activation of PI3K/Akt and MAPK/ERK signaling pathways and promote epithelial‐mesenchymal transition.^[^ ^88^ ^]^ The miRNA HBV‐miR‐3 can regulate the expression of *PPM1A*, a gene related to viral infection and HCC development.^[^ ^89^ ^]^ HPV16‐encoded miRNAs down‐regulate *ARID5B*, *ZEB2*, and *THBS1*, affecting cell migration, while also suppressing *STAT5B* and modulating JAK‐STAT and ErbB signaling pathways.^[^ ^90^ ^]^ RNA viruses also can encode v‐miRNAs; For example, HIV‐1 produced functional vsmiRNAs stemmed from the nef and env gene loci.^[^ ^91^ ^]^ Therefore, virus‐derived miRNAs can regulate host pathogenic genes and act as vital factors for disease progression.

### 7.5 Nucleic Acid Fragment as Pathogenic Elements Beyond Protein

As the functional executors, proteins are regarded as vital pathogenic substances in various RNA and DNA viruses. For RNA viruses, NSP8 and NSP9 encoded by SARS‐CoV‐2 bind to 7SL RNA and disturb host cell protein trafficking,^[^ ^92^ ^]^ enabling the virus to resist host defense system and enhance infection. In DNA viruses, the HBV X protein, expressed by HBV, induces degradation of Smc5/6 complex in hepatocytes and leads to DNA damage accumulation,^[^ ^93^ ^]^ which contributes to HBV‐induced hepatocellular carcinoma. Surprisingly, the HPV‐encoded E7 protein, despite consisting of only 100 amino acids, regulates the cell cycle and metastasis and is considered as oncoproteins in cervical cancer.^[^ ^94^ ^]^ Thus, these pathogenic proteins represent potential drug targets for relevant disease therapies.

Unlike proteins, the pathogenic potential of nucleotide sequences has rarely been discussed despite their fundamental role as carriers of genetic information. In our studies, we demonstrated that HISs of SARS‐CoV‐2 significantly triggers increased hyaluronan production in COVID‐19 patients and causes severe clinical symptoms including lymphocytopenia and pulmonary lesions.^[^ ^21^ ^]^ These pathological effects are efficiently ameliorated in patients following treatment with the hyaluronan inhibitor hymecromone.^[^ ^83^ ^]^ Similarly, the HISs of SARS‐CoV was found to promote the hyaluronan synthesis and accumulation in pulmonary cells,^[^ ^21^ ^]^ resulting in pulmonary ground‐glass opacity and consolidation in Severe Acute Respiratory Syndrome (SARS) patients. Excitingly, HBV‐SITEs are identified as another pathogenic element and act as oncogenic driver for HBV‐induced HCC in our recent study,^[^ ^80^ ^]^ which suggests that viral integrated sequence themselves are significant pathogenic substances for the disease. In line with these results, we also found that SITEs of HPV16 epigenetically facilitate the development of HPV‐induced cervical cancer. These findings collectively emphasize that nucleotide sequences can function as significant pathogenic factors beyond protein.

Therefore, we propose that nucleic acid fragments (such as miRNAs/HISs/SITEs) are crucial pathogenic elements in various diseases, including infectious diseases, and termed them as “**P**athogenic **N**ucleic **A**cid **S**equences” (PNAS). In this context, we can strategically develop nucleotide‐based drugs targeting PNAS for disease prevention and treatment. Notably, when designing mRNA vaccines, we must carefully avoid or mutate the PNAS in viral genomes, particularly when they are embedded within the coding sequences of pathogenic proteins; otherwise, the vaccine may induce serious side effects.

### 7.6 Targeting HISs or miRNAs as Effective Strategies for Virus‐related Disease Therapy

HISs are highly conserved in eight primates, including humans, rhesus monkeys, and cynomolgus monkeys, which is consistent with the susceptibility of primates to COVID‐19 and related clinical manifestations. While SARS‐CoV‐2 infection may lead to human death, it is not fatal to potential hosts such as bats and pangolins. This observation indicates that HISs may determine the species specificity of virus infection. Interestingly, HISs also exist in other viruses, including human coronavirus, HIV, and Ebola virus, which can target the specific organ and tissue enhancers to promote the pathogenic process of these viruses.

Targeting virus miRNA and regulating host enhancers may be effective treatments for virus‐related diseases. JQ1, an inhibitor of enhancer activity, can reduce the expression of genes activated by HISs, while Cas13d‐mediated HIS degradation effectively inhibits the activation of inflammatory genes induced by HISs. Meanwhile, hymecromone, a drug to inhibit hyaluronic acid synthesis, can reduce the hyaluronic acid accumulation caused by HISs. Treatment with hymecromone has been shown to promote the improvement of clinical symptoms in COVID‐19 patients, such as decreased lymphocyte count and lung lesions.^[^ ^83^ ^]^ Moreover, the inhibitor and antagomir of virus miRNA can inhibit the activity of the enhancer, thereby decreasing the target gene expression and blocking the disease progression of virus infection. For example, antagomir of HISs in SARS‐CoV‐2 can downregulate the expression of corresponding inflammatory genes, such as *KALRN* and *MYL9*.^[^ ^21^ ^]^ In addition, we have demonstrated that inhibitors targeting HBV‐miR‐2 in HBV‐SITE‐1 significantly inhibits HCC growth in vivo through decreasing the oncogenic gene expression.^[^ ^80^ ^]^ Therefore, targeting HISs or miRNAs could be an efficient strategy for fighting against RNA virus‐related diseases.

## 8 NamiRNA‐mediated Gene Activation Targeting Enhancer

**Figure 4:**
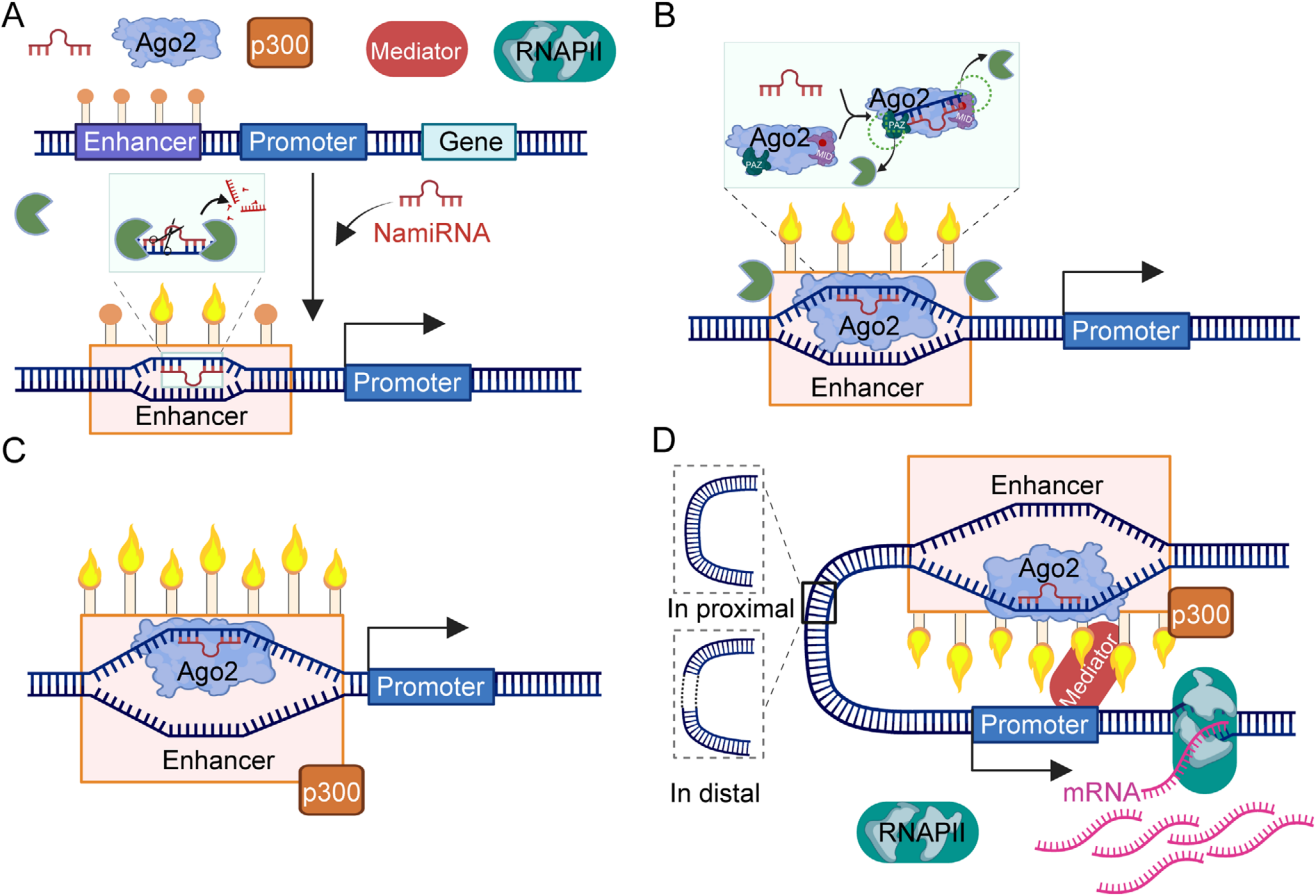
NamiRNA‐enhancer‐mediated gene activation model (created with permission by BioRender). A). NamiRNAs bind to the enhancer in a sequence‐dependent manner and initially open the double strands of DNA. However, the binding of NamiRNAs are partly degraded by cellular RNases (such as RNase H) without AGO2. B) AGO2 binding to miRNA results in a conformational change to confers RNase resistance. Specially, NamiRNA binds to the PAZ and MID domains of the AGO2 protein, which induces a conformational change in NamiRNA that protects it from cleavage by RNase. C) NamiRNA‐mediated enhancer activation. Ago2 facilitate the recruitment of histone‐modifying enzymes, including p300, to increase H3K27ac at enhancer regions. D) Activated enhancers caused by NamiRNAs interact with promoter to activate gene transcription through coordinated recruitment of RNA poly II and mediator complex.

Our studies have demonstrated that NamiRNAs function as epigenetic regulators. Through a series of molecular and genomic analyses, we have uncovered the regulatory pattern by which NamiRNAs activate gene expression, including enhancer activation, sequence‐specific miRNA‐enhancer interactions, AGO2 involvement, and R‐loop formation (**Figure** **4**). Accordingly, we propose the NamiRNA‐Enhancer‐mediated Gene Activation theory, which provides novel research perspectives and potential therapeutic strategies for understanding cellular proliferation or apoptosis, individual growth and development, and diseases like tumorigenesis.^[^ ^95^ ^]^ In detail, these miRNAs exhibit genomic colocalization with H3K27ac‐marked active enhancers and initiate transcriptional activation through a multi‐step process: (1) Sequence‐dependent binding to enhancer regions induces DNA duplex opening, although NamiRNAs are vulnerable to RNase H degradation without AGO2 protection; (2) AGO2 binding via its PAZ domain induces conformational changes that shield NamiRNAs from RNase cleavage while stabilizing enhancer interactions; (3) The AGO2‐NamiRNA complex recruits histone modifiers like p300 to deposit H3K27ac marks, altering chromatin accessibility; (4) Activated enhancers establish chromatin looping, recruiting transcriptional machinery (RNA Pol II and mediator complexes) to initiate transcription. These mechanistic insights shed new light on the non‐canonical regulatory roles of miRNAs and their implications in development and diseases.

### 8.1 NamiRNAs Act as Enhancer Triggers

Given the genomic co‐localization between miRNAs and enhancers, as well as the central role of RNA polymerase II in transcriptional regulation, we performed chromatin immunoprecipitation (ChIP) assays to examine the enrichment of Pol II, P300, and H3K27ac at the genomic locus of miR‐24‐1. Overexpression of miR‐24‐1 in HEK293T cells led to a significant enrichment of Pol II, P300, and H3K27ac at this locus, suggesting that miR‐24‐1 may promote enhancer activation by recruiting histone‐modifying enzymes such as P300, thereby enhancing the transcriptional activation of its target genes.^[^ ^14^ ^]^

Furthermore, RNA‐seq analysis revealed that the number of upregulated and downregulated genes upon miR‐24‐1 overexpression was approximately equal, indicating a broad impact on the transcriptome. Notably, ChIP‐seq data shows that ≈60% of the regions targeted by miR‐24‐1 are overlapped with enhancer elements.^[^ ^14^ ^]^ MiR‐339, as a NamiRNA, can activate the reporter gene by interacting with the enhancer in the luciferase activity experiment.^[^ ^15^ ^]^ The ratio of Luc+/Rluc+ in the experimental group is remarkably higher than that in the control group with co‐transfecting pGL3‐miR‐339 and pSUPER empty plasmids. Additionally, miR‐339 can increase the enrichment of H3K27ac in the enhancer region 60 kb upstream of *GPER1*, indicating miR‐339 is able to upregulate enhancer activity. Collectively, these results support a model in which enhancer‐associated miRNAs can globally upregulate gene expression at the transcriptomic level by specifically activating enhancer activity.

### 8.2 NamiRNAs Interact with the Enhancer on Sequence‐specific Matching

In our mechanistic studies of NamiRNAs, we demonstrated that the enhancer‐activating capacity of miR‐339 critically relies on sequence‐specific interactions between its “seed region” (nucleotides 2–8) and complementary motifs within target enhancers.^[^ ^15^ ^]^ To dissect this dependency, we performed “site‐directed mutagenesis” on the seed sequence of miR‐339 and its binding sites of the enhancer sequence, followed by dual‐luciferase reporter assays in HEK293T cells. Disruption of the miR‐339 seed sequence completely abrogated its ability to activate the wild‐type enhancer, reducing luciferase activity to baseline levels (*P* < 0.001 vs wild‐type miR‐339). This loss‐of‐function phenotype confirms that the seed region, rather than passenger sequences, mediates enhancer targeting. Conversely, mutating the enhancer's miR‐339‐binding motif (8‐nt complementary site) abolished activation by wild‐type miR‐339, with reporter activity indistinguishable from negative controls (*P* < 0.001).^[^ ^15^ ^]^

The mutual disruption of enhancer activation in both miRNA‐ and enhancer‐mutant scenarios highlights a “lock‐and‐key” mechanism governing NamiRNA functionality. Notably, neither mutant miR‐339 nor the mutant enhancer exhibited dominant‐negative effects when co‐transfected with their wild‐type counterparts, suggesting the interaction is highly specific and non‐competitive. These findings redefine NamiRNAs as sequence‐dependent epigenetic modulators, where seed integrity dictates their nuclear regulatory capacity, distinct from their cytoplasmic mRNA‐silencing roles. Such precision implies that therapeutic strategies targeting NamiRNAs must preserve or disrupt seed motifs to selectively modulate enhancer activity.

### 8.3 AGO2 in NamiRNA‐Mediated Gene Activation

AGO2 plays a pivotal role in NamiRNA‐mediated gene activation as a critical regulatory factor. Specifically, AGO2 forms stable complexes with NamiRNAs and their complementary single‐stranded DNA (ssDNA) hybrid strands at enhancer regions,^[^ ^18^ ^]^ thereby protecting these RNA‐DNA hybrids from degradation by RNase H. This protective mechanism enables sustained interactions between NamiRNAs and enhancers, facilitating transcriptional activation.

Structurally, AGO2 binds NamiRNAs via its PIWI‐AGO‐Zwille (PAZ) domain, while its PIWI domain recognizes and anchors the ssDNA hybrid strand. This dual binding not only stabilizes NamiRNAs in the nuclear compartment but also facilitates the recruitment of transcriptional co‐activators or chromatin remodeling complexes to enhance gene expression. Furthermore, AGO2 interacts with histone‐modifying enzymes such as p300, promoting H3K27ac deposition at enhancer regions, which amplifies enhancer activity.

In summary, AGO2 acts as a “guardian” in NamiRNA‐mediated transcriptional activation by preserving RNA‐DNA hybrid integrity and orchestrating chromatin‐modifying events. These findings redefine AGO2's non‐canonical nuclear functions and highlight its potential as a therapeutic target for disrupting oncogenic enhancer reprogramming in cancers.

### 8.4 NamiRNA‐Mediated R‐Loop Formation Promotes Gene Transcription

NamiRNAs drive gene transcription by facilitating the formation of R‐loops, three‐stranded nucleic acid structures composed of an RNA‐DNA hybrid (formed by the RNA strand and DNA template strand) and a displaced single‐stranded DNA non‐template strand. In the context of NamiRNA activity, R‐loops are selectively generated at enhancer regions, where they modulate local chromatin architecture to potentiate transcriptional activation.

Mechanistically, NamiRNAs hybridize with complementary DNA sequences at enhancers, recruiting RNA polymerase II (Poly II) and other transcriptional machinery components. During transcription, the nascent RNA strand generated by Pol II anneals to the DNA template strand, stabilizing the transcription bubble and promoting transcriptional elongation. Concurrently, R‐loop formation may recruit chromatin remodelers and histone‐modifying enzymes, which further enhance chromatin accessibility and transcriptional output.

Notably, R‐loops exert dual effects on genomic stability. In NamiRNA‐mediated transcriptional programs, R‐loops transiently enhance cellular adaptability by amplifying oncogenic gene expression. However, persistent R‐loops may interfere with DNA replication and repair processes, increasing susceptibility to DNA damage and mutagenesis, thereby creating a permissive environment for malignant transformation. Consequently, elucidating the molecular mechanisms of NamiRNA‐driven R‐loop formation (such as their context‐dependent roles in transcriptional activation versus genomic instability) is essential for unraveling carcinogenic pathways and developing strategies to selectively target R‐loop dynamics in disease therapy.

## 9 Research Roadmap of NamiRNA‐Enhancer Network

A major challenge in elucidating the NamiRNA‐enhancer network lies in delineating the functional connections between NamiRNAs, enhancer regions, and the downstream genes they potentially activate. This section summarizes key strategies for addressing these challenges.

### 9.1 How to Select Specific NamiRNAs and Cell Types?

Candidate NamiRNAs are differently expressed under specific pathological conditions, exhibit nuclear localization, and are associated with enhancer regions displaying transcriptional activation phenotypes.^[^ ^96^, ^97^ ^]^ Cell types appropriate for investigation often include transcriptionally active systems, such as tumor cells, stem cells, or activated immune cells. Public resources such as TCGA and ENCODE can be combined with experimental datasets to facilitate the preliminary bioinformatic screening of candidate NamiRNAs and their cellular contexts.^[^ ^98^ ^]^

### 9.2 How to Construct and Validate NamiRNA‐enhancer Networks?

Integration of multi‐omics data is used to assess whether a given miRNA exerts gene‐activating effects via enhancer elements. Specially, the active enhancer regions are identified through peak calling from H3K4me1 and/or H3K27ac ChIP‐seq data. And miRNA‐seq and mRNA‐seq (or array‐based profiling) are used to identify the differentially expressed miRNAs and mRNAs, forming the basis for putative positive miRNA‐gene regulatory pairs. And miRNAs binding sites within enhancer regions was predicted using tools like RNAhybrid or miRanda.^[^ ^99^, ^100^ ^]^ Subsequently, functional assays such as NamiRNA knockdown or CRISPR interference targeting enhancer regions are employed to validate whether target gene expression decreases following disruption, thereby confirming the directionality of activation. Additionally, clinical datasets can be utilized to evaluate the expression patterns and prognostic significance of target genes in specific diseases, including various cancers and immune‐related disorders. Based on these defined regulatory cascades, a pathway model of “NamiRNA → enhancer → targeted gene” is established.

### 9.3 What Are the Current Challenges in NamiRNA Research?

Despite the emerging importance of NamiRNAs as potential transcriptional activators, several key challenges remain. First, the lack of standardized criteria for identifying nuclear‐localized miRNAs hampers the systematic screening of candidate NamiRNAs.^[^ ^101^ ^]^ Second, the underlying mechanisms remain incompletely understood, particularly regarding the differences between AGO‐dependent and AGO‐independent activation pathways. Third, large‐scale functional validation and disease association studies are still limited. Therefore, we advocate for the establishment of a unified research framework that integrates subcellular localization, functional validation, and clinical relevance. Coupled with advances in high‐throughput omics technologies, mechanistic studies, and clinical data integration, NamiRNA research can evolve from fundamental discovery to translational application in precision medicine.

## 10 Reevaluating miRNA Biological Functions Through the Lens of NamiRNA‐Enhancer Network

The NamiRNA‐Enhancer‐mediated Gene Activation theory has been validated across multiple diseases,^[^ ^15^, ^16^, ^17^, ^18^ ^]^ revealing a novel gene regulatory pattern that reshapes our understanding of miRNA functions. Here, we cautiously summarize some important questions regarding miRNAs and provide an in‐depth discussion as follows.

### 10.1 Are miRNAs’ Functions Indeed Weak or Powerful?

As stable small non‐coding RNAs, miRNAs regulate critical physiological and pathological processes such as cell differentiation and tumorigenesis by modulation of gene expression. Notably, some miRNAs display clear tissue‐specific expression patterns. For example, miR‐1 and miR‐133 are mainly enriched in myocardial and skeletal muscle cells, contributing to muscle development,^[^ ^102^ ^]^ while miR‐122, as an important regulator of liver biological function, is highly expressed in the liver.^[^ ^103^ ^]^ In addition, the specific expression of miRNAs is closely associated with the occurrence and development of different tumors. For instance, miR‐31 and miR‐375 are significantly up‐regulated in the early stage of esophageal cancer,^[^ ^104^ ^]^ and miR‐129 is markedly elevated in gastric cancer.^[^ ^105^ ^]^ Collectively, these findings underscore the profound roles of miRNAs in the progression of development and diseases.

Recent studies have demonstrated that tissue‐specific miRNAs can determine cell types and cell fate. A typical example is miR‐122, a well‐known miRNA with high expression in the liver, which plays a key role in the development of the liver embryo, liver maintenance and tumorigenesis. During the embryonic development in mice, miR‐122 is continuously increased since it is detected in hepatocytes after 12.5 days, reaching a plateau before the mice are born, and it can still increase slowly after birth,^[^ ^106^ ^]^ indicating that miR‐122 is crucial for vital activities after the birth of mammals. In lines with this sight, knockout of miR‐122 directly destroys liver homeostasis and induces hepatocarcinogenesis when the male mice are 11 months.^[^ ^6^ ^]^ Besides, miR‐122 combined with miR‐148a, miR‐424, miR‐542‐5p, and miR‐1246 can rapidly and effectively induce mesenchymal stem cells into liver‐like cells, thus maintaining the characteristics of miR‐122 and liver cells.^[^ ^107^ ^]^ Our results demonstrated that miR‐122 is located at genomic enhancer regions and reactivates the liver‐specific genes *FOXA1*, *FOXA2*, *HNF4*, and *HNF6* in HepG2 cells, suggesting that miR‐122 determines the biological function of the liver through regulating liver‐specific transcription factors. In other words, low miR‐122 expression results in reduced liver‐specific gene expression and a loss of hepatocyte identity in hepatocellular carcinoma. Similarly, based on the NamiRNA‐enhancer network, most tissue‐specific miRNAs can take charge of regulation on tissue‐specific genes to determine cell fate transition. Therefore, miRNAs are indispensable components for sustaining the cell identity and organ function, especially after birth.

### 10.2 Does miRNA‐Mediated Gene Regulation Mainly Occur in the Cytoplasm or Nucleus?

It is well‐known that the subcellular localization of miRNAs determines their biological function. Numerous studies have revealed that miRNAs inhibit gene expression via targeting the UTRs of mRNAs in the cytoplasm, which is appointed as traditional miRNAs in our context. In recent years, it has been reported that miRNA also exists in the nucleus beyond the cytoplasm. For example, miR‐29b has a sequence similar to a nuclear localization signal and is enriched in the nuclei of HeLa and NIH 3T3 cells.^[^ ^96^ ^]^ Specially, we have demonstrated that NamiRNAs activate gene transcription by targeting the enhancer in the nucleus. So, which one is the more important gene regulatory pattern for biological function?

Strikingly, NamiRNAs and traditional miRNAs are not only closely connected but also have their own characteristics. Specifically, the sequence of NamiRNAs is the same as that of traditional miRNAs, but their subcellular localization and gene regulation patterns are significantly different. Based on the classical miRNA inhibition theory, the traditional miRNAs bind to the UTRs of mRNAs and inhibit their expression at the post‐transcriptional level. However, NamiRNAs are a class of miRNAs located in the nucleus that activate gene expression at the transcription level through interaction with enhancers, and its genomic position is highly overlapped with enhancer markers such as H3K4me1 or H3K27ac. Notably, we found that more than 1200 miRNAs overlap with enhancers, suggesting that most miRNAs have the potential for gene activation as NamiRNAs. Therefore, NamiRNAs and traditional miRNAs are both important component for miRNAs. In particular, the disorder of NamiRNA‐enhancer‐gene activation network is responsible for the loss of normal cell identity and transformation into tumor cells.

In our opinion, miRNAs can function as gene “repressors” and “activators” to coregulate the cell identity and their function. Specially, cytoplasmic miRNAs inhibit the expression of target genes in a classical way while the nuclear miRNAs activate gene expression by NamiRNA‐enhancer network. Here, we take the transformation of cell identity and tumorigenesis as a represent example. miRNAs are involved in maintaining the ten characteristics of tumor cells.^[^ ^108^ ^]^ The abnormally high‐expressed miRNAs interfere with the normal expression of their target genes by targeting the 3′ UTRs of mRNAs, which leads to normal cells acquiring the biological characteristics of cancer cells.^[^ ^109^ ^]^ On the contrary, some other miRNAs in tumor cells are decreased and show the low expression in tumor cells.^[^ ^15^, ^109^ ^]^ According to our research, many miRNAs with low expression in tumors, especially some common tumor suppressor miRNAs, such as hsa‐miR‐34, hsa‐miR‐200c, hsa‐let‐7a‐1, and miR‐339 are classified as NamiRNAs.^[^ ^14^ ^]^ For example, decrease in NamiR‐339 positively regulates the reduction of TSG expression.^[^ ^15^ ^]^ In this case, overexpression of these miRNAs leads to the high expression of TSG, thus restraining the growth and abnormal differentiation of tumor cells. In fact, when normal cells develop into tumor cells, the original cell identify and function have changed along with the abnormal expression of miRNAs. For example, knockout of miR‐122 results in the accumulation of lipids in liver^[^ ^6^ ^]^ while it is normal for adipose tissue to store lipids, suggesting that deletion of miR‐122 causes the loss of hepatocellular function. Therefore, the bidirectional regulation of genes by miRNAs is unified pathways to maintain cells or organs function well.

### 10.3 Are miRNAs Themselves Regulated by Promoters or Enhancers?

A longstanding question in the field of non‐coding RNA regulation is whether miRNAs require canonical promoters to initiate transcription or whether they can function independently as regulatory DNA elements. The conventional view posits that pri‐miRNAs are transcribed by RNA polymerase II from distinct promoter regions marked by defined transcription start sites and characteristic chromatin features.^[^ ^110^ ^]^ However, emerging evidence, particularly involving nuclear‐localized miRNAs, suggests that certain miRNAs exhibit enhancer‐like activity and can directly participate in long‐range gene activation.

Studies of NamiRNAs have demonstrated that these miRNAs physically interact with enhancer elements and function as integral components of enhancer complexes. Through association with RNA‐binding proteins such as AGO2 and recruitment of transcriptional machinery, NamiRNAs promote the production of enhancer RNAs and enhance the transcription of downstream target genes. Moreover, many miRNA families that share the same seed sequence but are transcribed from distinct genomic loci exhibit spatiotemporally distinct expression patterns. This modular regulatory behavior is often governed not by static promoter elements but by dynamic enhancer activation in response to developmental cues or environmental stimuli.^[^ ^111^ ^]^ For instance, the miR‐17–92 cluster and its paralogs regulate cell cycle progression in both embryonic development and cancer, yet their expression is driven by distinct enhancer contexts in hematopoietic versus epithelial tissues.^[^ ^112^, ^113^ ^]^

Taken together, these findings support a paradigm shift in which certain miRNAs should not be regarded merely as passive transcriptional outputs, but rather as active regulatory elements with enhancer‐like properties capable of modulating gene expression in trans. Future research should move beyond the promoter‐centric model of miRNA function and instead focus on defining the chromatin context, RNA‐protein interactions, and regulatory triplets (miRNA‐enhancer‐gene) that underpin their transcriptional activation capacities.

### 10.4 What's the Exact Function of AGO2 in miRNA‐Mediated Gene Regulation?

It is reported that AGO2 is mainly composed of three domains, including PAZ, Middle (MID), and PIWI.^[^ ^37^ ^]^ Interestingly, AGO2 could be located in both the cytoplasm and nucleus,^[^ ^18^, ^36^ ^]^ which function differentially in miRNA‐mediated gene regulation. For gene silencing by miRNAs,^[^ ^37^ ^]^ AGO2 in the cytoplasm recognizes the 5′ and 3′ end of miRNA via the binding pockets of PAZ and MID domains to form miRISC, which further induces the inhibition of translation or degradation of complementary target mRNAs. Instead, recent research has demonstrated that AGO2 participates in gene transcription by NamiRNAs in the nucleus.^[^ ^14^, ^15^, ^18^ ^]^

Specifically, AGO2 has dual functions for NamiRNA‐mediated gene activation. On the one hand, AGO2 binds the hybrids of NamiRNA‐Enhancer DNA dependent on the PAZ domain and protects them from the degradation of RNase H.^[^ ^18^ ^]^ Accordingly, AGO2 can maintain the stability of this duplex and maintain the double‐stranded DNA in an open state, which provides a suitable condition for gene transcription by RNA Pol II. On the other hand, there is a colocalization of AGO2 and P300 in NamiRNA‐mediated gene activation,^[^ ^18^ ^]^ indicating that AGO2 may act as an anchor protein to recruit histone‐modifying factors such as P300 and result in the enrichment of H3K27ac to activate enhancers. Therefore, AGO2 is a necessary modulator for NamiRNA‐mediated gene activation, and its detailed function deserves our efforts for investigation in the future.

### 10.5 How Do miRNAs Enter the Cell Nucleus and Function as NamiRNAs?

In eukaryotic cells, the spatial separation of nucleus and cytoplasm is accomplished by a double‐layer of membrane structure named nuclear membrane,^[^ ^114^ ^]^ which controls the transport of substances between nucleus and cytoplasm. Generally, genomic miRNAs are transcribed into pri‐miRNAs in the nucleus, processed into pre‐miRNA, and then exported to the cytoplasm for the mature miRNA. However, how mature miRNAs localize to the nucleus remains unclear.

It is reported that the nuclear localization of miR‐29b is contributable for its sequential similarity to a nuclear localization sequence,^[^ ^115^ ^]^ suggesting that specific features of miRNA sequences could serve as a sign for the nuclear entry for miRNAs. In fact, the nuclear membrane undergoes a dynamic process involving reconstruction after disappearance during the cell cycle.^[^ ^114^ ^]^ Notably, the nuclear membrane is completely invisible at the M phase of the cell cycle, which provides a convenience for the substance exchange between the cytoplasm and nucleus, potentially allowing miRNA nuclear import. Besides, there may be some unknown pathways for miRNA biogenesis that the pre‐miRNA could be processed into matured miRNAs in the nucleus without exporting to the cytoplasm and back into the nucleus.

### 10.6 Are Tissue‐specific miRNAs as Epigenetic Orchestrators for Organ Function?

Given the shared tissue‐specific features of miRNAs and enhancers, tissue‐specific miRNAs could serve as NamiRNAs. In our opinion, tissue‐specific NamiRNA signatures are as critical determinants of organ development and physiological function, fine‐tuning transcriptional networks through direct mRNA targeting and enhancer interactions. These functional paradigms are illustrated by four prominent tissue‐specific miRNAs in hepatic, muscular, neural, and immune systems.

#### 10.6.1 Hepatic miR‐122: Liver‐Enriched Metabolic Regulator

Liver‐specific miR‐122 serves as a cruciual regulator in hepatocellular carcinoma pathogenesis. We found that marked downregulation of miR‐122 in HCC tissues can activate hepato‐specific genes like *FOXA1* and *HNF4* through interaction with enhancers. miR‐122 depletion silences hepatocyte identity genes, triggering dedifferentiation and malignant transformation. Functional studies reveal that exogenous miR‐122 restoration reactivates hepatic phenotype genes while suppressing HCC cell proliferation and metastasis. Mechanistically, miR‐122 employs AGO2‐mediated enhancer RNA recruitment to stabilize liver‐specific transcriptional complexes, thereby maintaining hepatocyte functional homeostasis. Preclinical models validate that miR‐122 deficiency accelerates HCC progression, whereas targeted delivery of miR‐122 agomirs significantly attenuates tumor growth. Notably, although miR‐122 is reported to strengthen HCV replication cycles,^[^ ^116^ ^]^ blocking miR‐122 function with inhibitors may accelerate the hepatocellular carcinoma progression due to the loss of liver cell identity caused by miR‐122 deficiency. These discoveries underscore the therapeutic potential of miR‐122 as a HCC target and provide a foundation for developing epigenetic intervention strategies.

#### 10.6.2 Muscle‐Specific miR‐1/133 Cluster: Architect of Myogenesis

The muscle‐specific miR‐1/133 bicistronic cluster plays a pivotal role as a master architect orchestrating the intricate process of myogenesis, which involves the formation and development of skeletal muscle. This cluster, comprising miR‐1 and miR‐133, acts as a cardinal regulator, fine‐tuning gene expression to ensure appropriate muscle cell differentiation and function.^[^ ^117^, ^118^ ^]^ miR‐1 facilitates myoblast differentiation by inhibiting histone deacetylase 4 (HDAC4), thereby relieving transcriptional repression of muscle‐specific genes and promoting their expression.^[^ ^119^, ^120^ ^]^ Concurrently, miR‐133 functions as a molecular switch, governing fiber‐type specification by targeting serum response factor, which is crucial for determining muscle fiber characteristics.^[^ ^119^ ^]^ Together, miR‐1 and miR‐133 orchestrate a harmonious balance, driving skeletal muscle development with precision and finesse.

#### 10.6.3 Neuronal miR‐124 and miR‐128: Guardian of Neurogenesis

Highly enriched in central nervous system neurons, miR‐128 exemplifies miRNA‐mediated RNA activation through direct enhancer binding. Our data also revealed that miR‐128 exhibits notably high expression in brain tissues and interacts with SQSTM1/p62 to maintain neuronal health through modulating intricate molecular pathways, including autophagy and protein homeostasis. Emerging research highlights its association with neurodegenerative disorders, where dysregulated miR‐128 levels correlate with disease progression. By targeting these pathways, miR‐128 could mitigate neurotoxic protein aggregation and synaptic dysfunction, offering a potential therapeutic strategy to slow neurodegeneration.

#### 10.6.4 Lymphocyte‐specific miR‐155: Supervisor of Immune

As an ancient regulator, miR‐155 is mainly expressed in the immune cells,^[^ ^121^ ^]^ especially in lymphocytes.^[^ ^122^ ^]^ The deficiency of miR‐155 disrupts normal immune function in mice, leading to immune deficiency.^[^ ^123^ ^]^ According to the UCSC Genome Browse, miR‐155 shows complementarity with long non‐coding RNA miR155HG in both human and mouse genomes, suggesting the potential regulation of miR‐155 on miR155HG through the NamiRNA‐enhancer network. Notably, in inflamed antigen‐presenting cells, highly expressed miR144HG encodes miPEP15, a 17‐amino acid micropeptide that modulates antigen presentation.^[^ ^124^ ^]^ These findings indicate miR‐155 may regulate antigen presentation by controlling miPEP155 expression. Thus, targeting miR‐155/miR155HG/miPEP15 axis represents a potential therapeutic strategy for immune function restoration.

## 11 Promising Perspective and Application of NamiRNAs

**Figure 5:**
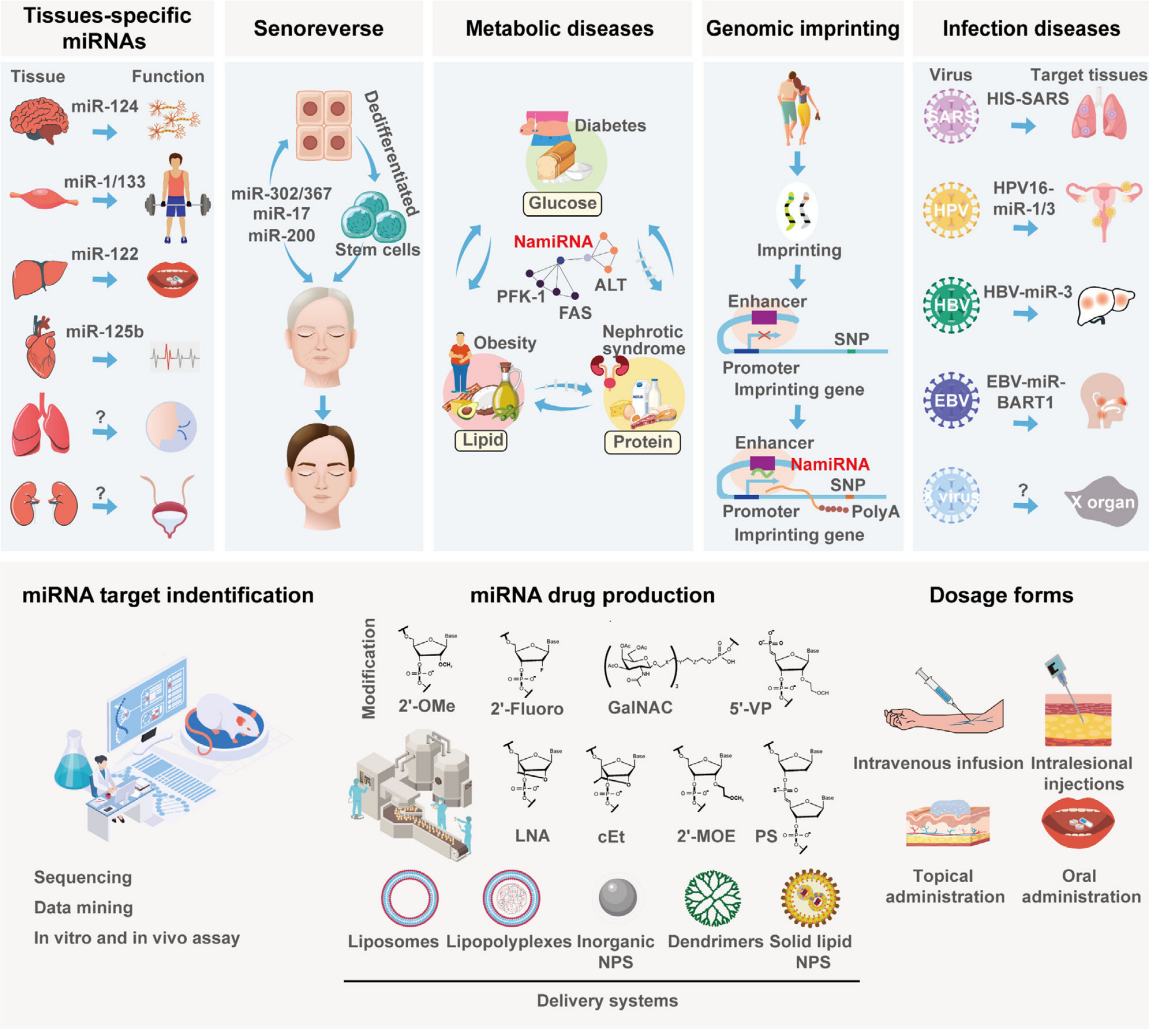
Potential application of NamiRNA/HIS (created with permission by Freepik). Tissue‐specific miRNAs function as key epigenetic regulators that maintain organ‐specific physiological homeostasis. Their regulatory roles highlight broad therapeutic potential in aging, metabolic diseases, genomic imprinting disorders, and infectious diseases. Advances in the development pipeline of NamiRNA‐based therapeutics support their clinical translation. PFK‐1, phosphofructokinase 1. ALT, alanine transaminase. FAS, fatty acid synthase. SNP, single‐nucleotide polymorphism. HIS, Human Identical Sequences. SARS, severe acute respiratory syndrome. HPV, human papillomavirus. HBV, hepatitis B virus. EBV, Epstein‐Barr virus. GalNAc, N‐acetylgalactosamine. 2′‐Ome, 2′‐O‐methyl. 5′‐VP, 5′‐vinylphosphonate. LNA, locked nucleic acid. cEt, constrained ethyl. 2′‐MOE, 2′‐O‐Methoxyethyl. PS, phosphorothioate backbone. NPs, inorganic nanoparticles.

The field of miRNAs has experienced significant advancements in recent years, unraveling their critical roles in various physiological and pathological processes. From orchestrating tissue‐specific functions to influencing stem cell dynamics, metabolic homeostasis, genomic imprinting, and host‐virus interactions, miRNAs have emerged as versatile regulators of both cellular and organismal biology, as well as innovative therapeutic targets. The subsequent sections delve into the multifaceted roles of miRNAs in these contexts and explore their potential as small nucleotide drugs (**Figure** **5**), highlighting the latest developments and future directions in miRNA research and clinical applications.

### 11.1 Stem‐Cell‐Specific miRNAs and Senoreverse

Aging is driven by systemic alterations, including cellular senescence, genomic instability, epigenetic dysregulation, loss of proteostasis, stem cell exhaustion, chronic inflammation, and impaired intercellular communication.^[^ ^125^ ^]^ These cumulative changes compromise physiological integrity, heighten vulnerability to age‐related diseases, and ultimately lead to mortality. Stem cell–specific miRNAs exhibit either exclusive expression in pluripotent stem cells or broad expression followed by rapid downregulation upon differentiation.^[^ ^126^ ^]^ These miRNAs critically regulate self‐renewal and multilineage differentiation, thereby orchestrating developmental processes and maintaining tissue homeostasis.

In human SCs, the predominant miRNA populations originate from a limited set of polycistronic loci encoding miRNAs with conserved seed sequences, including miR‐302–367, miR‐17–92, miR‐371–373, miR‐200, and the chromosome 19 miRNA cluster. Among them, the miR‐302 cluster enhances somatic cell reprogramming efficiency during iPSC generation.^[^ ^127^, ^128^ ^]^ Beyond miR‐302b, other stem cell‐related miRNAs also show age‐related relevance. The miR‐17 cluster is downregulated in human aging.^[^ ^129^ ^]^ Similarly, several members of the miR‐200 family are reported to reduce senescence marker expression and attenuate cellular senescence.^[^ ^130^, ^131^ ^]^ Strikingly, we found that miR‐302 can upregulate SOX2 and KLF4 expression in human fibroblasts to support pluripotency maintenance. In line with this insight, miR‐302b exhibits potent anti‐aging properties, effectively reversing senescence markers in vitro and restoring cellular and organismal function in aged mice by targeting *Cdkn1* and *CCng2* genes to facilitate the G1‐to‐S phase transition.^[^ ^132^ ^]^ Interestingly, systemic delivery of miR‐302b markedly reduced circulating pro‐inflammatory cytokines (IL‐1β, IFN‐γ, IL‐6, TNF‐α) and ameliorated chronic low‐grade inflammation in aged mice.^[^ ^132^ ^]^ Crucially, long‐term administration of miR‐302b over 25 months results in significant functional improvements and exhibits a favorable safety profile.

Therefore, stem cell‐specific miRNAs (such as miR‐302b, miR‐17, and miR‐200 family) represent a promising class of regulators in the context of aging. Their capacity to restore cellular function and systemic homeostasis underscores their therapeutic potential and paves the way for innovative miRNA‐based strategies to combat aging and age‐related diseases.

### 11.2 MiRNAs and Metabolic Regulation

Growing evidence has revealed that miRNAs are important regulators of balancing individual metabolic homeostasis and their dysregulation is closely associated with different metabolic diseases, including non‐alcoholic fatty liver diseases (NAFLD) and obesity.^[^ ^9^, ^133^, ^134^ ^]^ For instance, liver‐specific miR‐122 is significantly elevated in patients with NAFLD,^[^ ^135^ ^]^ indicating the upregulation of miR‐122 may contribute to lipid accumulation in the liver. Our results show that overexpression of miR‐122 could activate the genes of enzymes (such as *Scd1*) involved in fat synthesis, suggesting that restoration of miR‐122 expression into nomal levels could be used to alleviate NAFLD. Fundamentally, the metabolism of glucose, lipids, and proteins is systematically orchestrated through diverse enzymatic reactions that generate necessary energy for maintaining vital activities. Based on the NamiRNA‐enhancer network, we can select potential NamiRNAs targeting the vital biological enzymes for the metabolism of glucose, lipids, and proteins, providing us a promising approach for the therapy of metabolic diseases.

### 11.3 MiRNAs and Genomic Imprinting

Genomic imprinting is a fascinating epigenetic phenomenon that a gene inherited from one of the parents is specifically marked (imprinted) in certain cells and only expressed in a monoallelic pattern. Current research has revealed that silenced genes in genomic imprinting are mainly attributed to the DNA methylation in Imprinting Control Regions (ICRs)^[^ ^136^, ^137^, ^138^ ^]^ or dependent on H3K27me3 modifications.^[^ ^139^, ^140^ ^]^ For instance, the ICR of the Dlk1‐Dio3 locus presents a hypermethylated state on the paternal allele, resulting in the silencing of imprinted genes.^[^ ^138^ ^]^ However, it seems a scientific problem for us to explore the regulatory mechanism of expressed genes in genomic imprinting. Our data demonstrated that miR‐382 within the Dlk1‐Dio3 locus can activate the expression of imprinted gene *RTL1*. Additionally, another imprinted gene, *ZNF597*, is upregulated by miR‐6126 in non‐imprinting regions. Consequently, miRNAs in different genomic regions can activate the expression of imprinted genes, providing a novel regulatory mechanism in genomic imprinting.

### 11.4 MiRNAs/HISs and Infectious Diseases

The Virus‐encoded miRNAs are key players in pathogenesis. Since the initial discovery of virus‐encoded miRNA in EBV,^[^ ^141^ ^]^ hundreds of such miRNAs have been documented.^[^ ^142^ ^]^ These viral miRNAs can not only regulate the expression of viral genes but also influence the interaction between the host and virus, thereby modulating the host response to infection. For example, EBV‐derived miR‐BART9 can promote cell migration by targeting the membrane protein E‐cadherin, resulting in the nasopharyngeal carcinoma cells to the characteristics of mesenchymal cells.^[^ ^143^ ^]^ In addition, HBV‐miR‐3 has been proved to regulate the expression of host *PPM1A* gene, thus promoting the progression of hepatocellular carcinoma.^[^ ^144^ ^]^ Concurrently, HBV‐miR‐3 can down‐regulate the host *SOCS5* gene and enhance the antiviral effects of the host immune system.^[^ ^145^ ^]^ In the study of HPV encoded miRNA, HPV16‐ miR‐1 targets *ARID5B* to affect cell migration, while HPV16‐miR‐3 targets *PDE1B* to affect cell apoptosis.^[^ ^90^ ^]^ Collectively, these findings underscore the significant role of vsmiRNAs in promoting the development of virus‐related cancers.

Additionally, we found that HISs of SARS‐CoV‐2 can induce cytokine storm and enhance the SARS‐CoV‐2 infection by activating the corresponding genes.^[^ ^21^ ^]^ Such HIS elements also exist in other viruses, including HIV and Ebola virus, which can target the specific tissue enhancers to promote the viral pathogenic processes. For instance, HISs of HIV1 can target the T cell enhancers to promote their apoptosis through upregulating apoptosis‐related genes, thereby aggravating the progression of AIDS. Similarly, Ebola virus‐derived HISs are overlapped with host enhancer regions, whose neighbor genes are related to the disease progression of Ebola virus infection. Therefore, HISs from RNA viruses that interact with the host‐specific enhancers may provide new insights into the viral pathogenicity and their infection specificity of specifical tissues and organs.

Given that the negative regulatory theory of miRNAs as “Fine Tune” has deeply rooted in most researchers, it seems impossible to develop the clinical drugs for infectious diseases targeting vsRNAs. However, based on the theory of NamiRNA‐Enhancer‐mediated Gene Activation, viral miRNAs and HISs can regulate the expression of host cell genes at the global genome level by activating host enhancers, which could be clinically effective drug targets for infectious diseases.

### 11.5 MiRNAs and Tumor Progression

Numerous studies on tumorigenesis have demonstrated that miRNAs significantly modulate the development and progression of various tumors.^[^ ^16^, ^17^, ^18^, ^146^ ^]^ Based on abundant evidence from previous studies and our own research, we propose that **L**oss **O**f and **G**ain **O**f (LOGO) Cell Identity constitutes indispensable initiating events in tumorigenesis and metastasis. During these processes, the NamiRNA‐enhancer‐mediated gene activation network systematically orchestrates the alteration of cell identity. In tumor initiation, a significant reduction of specific miRNAs leads to the downregulation of genes that maintain original cell identity, contributing to the transformation of normal cells into cancerous cells. For tumor metastasis, cancerous cells require the acquisition of specific miRNAs to activate a set of genes that maintain target tissue cellular identity. Here, we use hepatic carcinoma as a representative example to discuss LOGO hypothesis in tumorigenesis. Specifically, liver‐specific miR‐122 is significantly downregulated in hepatocarcinogenesis,^[^ ^6^ ^]^ resulting in the decreased expression of liver‐specific genes (such as *HNF4*) via NamiRNA‐enhancer network and promoting the loss of hepatic cell identity during the hepatoma initiation. Notably, hepatoma metastases preferentially target the lungs. Given that the LM3 cell line (derived from 97L) exhibits high lung metastatic potential, we analyzed the distribution of H3K27ac enhancer markers between these cell lines and liver tissue. Surprisingly, 97L gains specific enhancers regulating lung development genes,^[^ ^147^ ^]^ which may be activated by lung‐specific miRNAs through the NamiRNA‐enhancer network. Thus, NamiRNAs represent promising therapeutic targets for reversing cell identity changes in both tumor initiation and metastasis, potentially enabling novel approaches for cancer treatment.

### 11.6 Targeting miRNAs for Developing Small Nucleotide Therapy

There are more than 138000 literatures on miRNAs recorded in PubMed. In recent years, numerous studies have focused on the clinical application of miRNAs as small nucleotide drugs. Based on the existing evidence and our research, several aspects of the miRNAs in clinical applications warrant attention, including the identification of therapeutic targets, optimization of production processes, and exploration of dosage forms.

#### 11.6.1 Identification of the Therapeutic Targets

It is well‐known that nucleotide drugs targeting miRNAs are typically developed in two forms: miRNA mimics and inhibitors. Generally, miRNA mimics can compensate for the functional deficiencies of their target miRNAs. Instead, miRNA inhibitors can suppress the function of these target miRNAs. Numerous excellent reviews have highlighted the promising clinical prospects of miRNA‐based therapeutics for treating diverse diseases.^[^ ^148^, ^149^, ^150^, ^151^ ^]^ In this context, a critical issue is how to identify the target miRNAs that exert crucial effects for the initiation and progression of diseases.

Typically, the differential expression of miRNAs and their target genes associated with the pathological processes of diseases serves as a primary indicator of their potential. This can be achieved through advances in the next‐generation sequencing techniques, such as Single‐cell miRNomics^[^ ^152^ ^]^ and Spatial miRNomics.^[^ ^153^ ^]^ Currently, several publicly available databases provide genomic and proteomic profiles from both healthy and diseased tissues, facilitating the large‐scale selection of miRNAs across different diseases.^[^ ^154^, ^155^, ^156^ ^]^ Subsequently, gain‐of‐function and loss‐of‐function experiments are used to validate the functional relevance of selected miRNAs using cell and tissue culture models, as well as in vivo systems. These approaches provide critical insights into the biological roles of candidate miRNAs and their potential therapeutic applications. Importantly, we can predict the potential impacts of miRNAs on different organs based on the NamiRNA‐enhancer gene network before their development into nucleotide drugs, which may significantly reduce side effects in clinical trials. Accordingly, we can construct extensive regulatory NamiRNA‐enhancer‐gene networks involving various diseases to expedite the development of nucleotide drugs in the future.

#### 11.6.2 Optimization of the Pharmaceutical Manufacturing Process

The production technologies are critical for maintaining the stability and effectiveness of miRNA therapeutics during large‐scale production. Several key manufacturing strategies can improve the stability, bioavailability, and overall therapeutic performance of miRNA‐based drugs. First, novel chemical modifications at the nucleotides can be introduced to enhance their stability and binding affinity.^[^ ^157^ ^]^ Meanwhile, several delivery platforms, such as GalNAc,^[^ ^158^ ^]^ can be employed in miRNA‐based drugs to enhance their cellular uptake, improve biodistribution, and facilitate endosomal escape. Additionally, optimizing other factors such as the nature of the carrier material, particle size, and surface charge is essential to protect the payload from degradation and ensure sustained release.^[^ ^159^ ^]^ Undoubtedly, consideration of these factors collectively will maximize therapeutic efficacy and facilitate the translation of miRNA‐based drugs into clinical applications.

#### 11.6.3 Exploration of the Dosage Forms

In general, the dosage forms of drugs are classified into solid and liquid states, affecting their administration routes. For liquid forms, both intravenous infusion and subcutaneous injection of miRNA‐based nucleotide drugs have been investigated as clinically viable options. For instance, intravenous administration of MRX34 with a controlled infusion pump resulted in a dose‐dependent change in gene expression in patients with tumors.^[^ ^160^ ^]^ Besides, subcutaneous injection of the sterile solutions of RG‐101 targeting miR‐122 successfully reduces the viral load in all treated patients within four weeks.^[^ ^161^ ^]^ Notably, local intradermal injection of Remlarsen (a miR‐29b mimic) in a hydrogel formulation inhibits the development of fibroplasia by reducing collagen expression in the injured skin of healthy volunteers.^[^ ^162^ ^]^ However, there are little clinical trials regarding the oral administration of miRNA‐based drugs, which deserve our investigation in the future. Furthermore, we could leverage the combination of appropriate dosage forms and administration routes to optimize the pharmacokinetic profiles and therapeutic effects of nucleotide drugs for various disease indications.

## 12 Concluding Remarks

In summary, miRNAs have emerged as critical regulators of gene expression across diverse physiological and pathological contexts, highlighting their potential as therapeutic targets. However, the development of miRNA‐targeting nucleotide drugs faces challenges, primarily due to the limited specificity in miRNA targets and the incomplete understanding of their mechanisms of action. This mechanistic ambiguity poses a significant hurdle for drug development, as a clear understanding of miRNA function is essential for designing effective therapeutics.

How should we interpret the seemingly paradoxical roles of miRNA‐mediated gene regulation in both the cytoplasm and nucleus? In our view, miRNAs function as critical mediators in cell identity transformation by bidirectional regulation of gene expression. Specifically, during the conversion of A cells to B cells, nuclear‐localized miRNAs activate B‐specific gene expression via the NamiRNA‐enhancer network, while their cytoplasmic counterparts silence A‐specific genes by targeting the UTRs of corresponding mRNAs. For instance, muscle‐specific miR‐1 and miR‐133 surppress non‐muscle gene expression (e.g., *Dll‐1*, a stemness‐associated factor) during ES cell differentiation.^[^ ^163^, ^164^ ^]^ Conversely, their nuclear counterparts could promote the transcription of muscle‐specific genes by binding enhancers. Collectively, these mechanisms highlight that cell identity could be theoretically manipulated through combinatorial miRNAs in a targeted manner.

What is the basic function of miRNAs in organismal development and pathological processes? Unlike key genes involved in embryonic development, knockout of most miRNAs (except for miR‐1) rarely leads to embryonic lethality or developmental defects, indicating that miRNAs are not essential for embryogenesis. In other words, miRNA‐mediated gene regulation primarily functions during post‐birth stages of lifespan. Although different organs have been progressively formed during pregnancy, some of them (such as the lung, stomach, and liver) do not perform the actual functions as they do after birth. We propose that miRNAs (especially tissue‐specific miRNAs) mainly regulate organ funtion through the NamiRNA‐enhancer network when individuals need to face the complicated post‐natal environment. In line with this insight, liver‐specific miR‐122 reaches a relatively high and stable level at birth, supporting hepatic metabolic function postnatally. Undoubtedly, abnormal miRNA expression may disrupt the normal function of organs and cause severe diseases. Therefore, miRNAs act as vital modulators of organ function and can be therapeutic targets to restore normal organ function in cases of developmental abnormalities or diseases.

The NamiRNA‐enhancer network provides a promising avenue to overcome different challenges, including the selection of candidate miRNAs and their target genes by precisely identifying and activating functional gene networks via miRNAs. For instance, specific miRNAs such as miR‐339 can activate a series of functionally similar tumor suppressor genes, including *GPER1*, while miR‐122 can restore the expression of hepatic‐specific genes such as *HNF4* and *HNF6*. This mechanism‐driven approach allows for the targeted modulation of multiple genes within a pathway, enhancing therapeutic efficacy and reducing off‐target effects.

By focusing on the regulatory functions of NamiRNAs, we can gain deeper insights into how miRNAs orchestrate gene expression dynamics, facilitating the development of more precise and effective miRNA‐based therapeutics. This approach not only deciphers complex regulatory networks underlying cellular processes but also enhances the therapeutic potential of targeting miRNAs. As research continues to unravel the intricate mechanisms of miRNA function, the future of miRNA‐targeting nucleotide drugs grows increasingly promising, holding the potential to revolutionize the treatment of a wide range of diseases.

## Conflict of Interest

The authors declare no conflict of interest.

## Author Contributions

Wenqiang Yu and Shuai Yang conceptualized the manuscript. Shuai Yang, Lu Chen, Ying Liang, Hongyan Wei, Mengxing Liu, Jin Wu, Wei Li, Wenxuan Li, Yuxiao Jin, Yinshan Li, Wei Zhao, Min Xiao, and Kaicheng Zhou wrote the draft of the manuscript. Shuai Yang, Hongyan Wei, Ying Liang, Jin Wu, Mengxing Liu, and Wei Li prepared the figures. Wenqiang Yu, Shuai Yang, Lu Chen, and Ying Liang mainly reviewed and revised the manuscript. All authors approved the final manuscript.

## Data Availability

The data that support the findings of this study are available from the corresponding author upon reasonable request.
